# Identification of the microglia-associated signature in experimental autoimmune encephalomyelitis

**DOI:** 10.3389/fimmu.2025.1581878

**Published:** 2025-06-05

**Authors:** Yan Wu, Jianhong Wang, Bo Chen, Yuxue Guo, Ping Gan, Yanbing Han

**Affiliations:** ^1^ Neurology Department, First Affiliated Hospital of Kunming Medical University, Kunming, China; ^2^ Kunming Medical University, Kunming, China; ^3^ Department of Neurology, Tongji Hospital of Tongji Medical College, Huazhong University of Science of Technology, Wuhan, China; ^4^ Biochemistry and Molecular Department, College of Basic Medicine, Kunming Medical University, Kunming, Yunnan, China

**Keywords:** RNA sequencing, microglia, multiple sclerosis (MS), experimental autoimmune encephalomyelitis (EAE), machine learning (ML)

## Abstract

**Background:**

Multiple sclerosis (MS) is a chronic neuroinflammatory disorder characterized by demyelination and immune dysregulation, and microglia play a central role in disease progression. Despite this, the specific microglial gene signatures contributing to MS remain inadequately characterized.

**Methods:**

We utilized an experimental autoimmune encephalomyelitis (EAE) mouse model and performed RNA sequencing to identify differentially expressed Messenger RNAs (DEmRNAs), Long Non-Coding RNAs (DElncRNAs), Circular RNAs (DEcircRNAs), and microRNAs (DEmiRNAs) in microglia. A machine learning approach incorporating five distinct algorithms was applied to select a robust multigene signature. The biological functions of the included genes were assessed through Gene Ontology (GO) and Kyoto Encyclopedia of Genes and Genomes (KEGG) enrichment analyses and validated by quantitative reverse transcription PCR (qRT-PCR). Additionally, molecular docking studies were conducted to explore potential interactions with approved MS therapeutics.

**Results:**

Six DEmRNAs were identified as key microglia-associated biomarkers: Neutrophilic Granule Protein (*NGP*), Histone Cluster 1 H2B Family Member J (*HIST1H2BJ*), Phenazine Biosynthesis-Like Domain-Containing Protein 1 (*PBLD1*), Muscleblind-Like Protein 3 (*MBNL3*), Lymphocyte Antigen 180 (*CD180*), and Coagulation Factor X (F10). All six genes were found to be upregulated in EAE microglia compared to phosphate-buffered saline (PBS) treated mice. These genes are primarily involved in immune-related pathways, including Toll-like receptor (TLR) signaling, and interact with MS therapeutics such as teriflunomide. Among the identified DEcircRNAs, circGAS2 (*mmu-circ-0001569*) was significantly upregulated, suggesting its potential regulatory role in microglial function. The expression trends of these biomarkers were validated via quantitative reverse transcription PCR (qRT-PCR) and Western blot analysis.

**Conclusions:**

This study provides a comprehensive microglial gene signature for EAE, highlighting the involvement of TLR pathways and circRNA-mediated regulation in MS pathogenesis. These findings provide a foundation for future research into microglia-targeted therapies and diagnostic tools for MS.

## Introduction

Multiple sclerosis (MS) is a chronic neuroinflammatory disease characterized by demyelination and neuronal damage that results in significant neurological disability (1). Microglia, the resident immune cells of the central nervous system (CNS), are central to MS pathology, as they contribute to both neuroinflammation and repair processes (2, 3). Emerging evidence highlights the complexity of microglial activation states, which are shaped by interactions with infiltrating immune cells and the local microenvironment (4–7).

Experimental autoimmune encephalomyelitis (EAE), the gold-standard preclinical model for studying immune-mediated neuroinflammation in MS (6), was selected for its unique ability to recapitulate adaptive immune-driven demyelination and microglial-immune crosstalk, features absent in toxin-induced models (e.g., cuprizone) (7). In this model, microglia transition through three critical phases. During the early induction phase (days 0–12 post-immunization), microglia initiate immune surveillance via proliferation and mitochondrial oxidative stress, priming the CNS for inflammation (8, 9). The acute inflammatory phase (peak stage, days 12–20) features peak demyelination driven by reactive oxygen species (ROS) bursts and M1-polarized (classically activated) microglial polarization, with CX3CR1-CX3CL1 axis-mediated leukocyte infiltration (10, 11). However, the chronic phase (days 20+) most closely models progressive MS, as microglia exhibit dynamic phenotype switching: TREM2/APOE-mediated lipid clearance and P2Y12R-PI3K/Akt signaling promote repair, while P2X7R-NLRP3 inflammasome activation perpetuates tumor necrosis factor-α (TNF-α) and interleukin-1β (IL-1β)/IL-18 secretion and chronic inflammation (5, 11–13).

At the transcriptomic level, RNA sequencing studies have uncovered profound heterogeneity in microglial activation states across MS lesions. In chronic active MS lesions, microglia adopt distinct transcriptional profiles, including lipid-phagocytic and iron-retentive subsets, marked by upregulated *TREM2, APOE*, and Complement C1q (*C1Q*) genes, which drive lipid metabolism, complement activation, and interferon responses (6, 8). In normal-appearing white matter, microglia exhibit pre-lesional activation, with upregulated glycolysis and iron homeostasis genes in gray matter and lipid metabolism genes in white matter, mirroring lesion-specific pathology (1, 14). Integration of transcriptomic data from the Gene Expression Omnibus (GEO) highlights immune-related pathways (e.g., JAK-STAT, PI3K-Akt) (15, 16) and identifies hub genes including *IL17A, STAT3* and *CXCR4* as critical regulators of neuroinflammation and remyelination failure (15, 17, 18). These findings underscore the dynamic transcriptional landscape of microglia in MS, offering potential biomarkers and therapeutic targets.

Emerging evidence highlights the pivotal role of non-coding RNAs (ncRNAs) in modulating immune crosstalk during EAE. Circular RNAs (circRNAs), such as circ_0000518 act as miRNA sponges to exacerbates M1 polarization of microglia/macrophages via the FUS/CaMKKβ/AMPK pathway (19). Similarly, long non-coding RNAs (lncRNAs), such as *NEAT1*, *KCNQ1OT1*, and miRNAs orchestrate adaptive immune responses by targeting key pathways (20). Furthermore, epitranscriptomic modifications, such as APOBEC/ADAR-mediated RNA editing, are significantly reduced during EAE progression, impairing microglial anti-inflammatory responses and worsening axonal damage (21). These ncRNA networks not only drive pathogenic immune cell polarization but also offer therapeutic avenues, as demonstrated by EphB3 inhibitors suppressing astrocyte-microglia interactions and ameliorating EAE (22). Collectively, RNA crosstalk represents a critical layer of regulation in autoimmune encephalomyelitis, linking genetic susceptibility to inflammatory neurodegeneration.

In this study, we employed RNA sequencing and machine learning (ML) approaches to identify microglial gene signatures and explore the differential expression of non-coding RNA, including circRNAs and lncRNAs, in the peak stage of EAE. Through differential expression analysis, functional enrichment, and molecular docking studies, we sought to uncover potential biomarkers and regulatory pathways relevant to MS. Additionally, we validated the expression of key biomarkers via both quantitative real-time PCR (qRT–PCR) and Western blot analysis, and explored their interactions with existing MS therapies. Our findings provide a foundation for future research into the molecular mechanisms of microglia in MS and their therapeutic targeting.

## Materials and methods

### Animals and disease modeling

Female C57BL/6 mice (n = 16, 8 weeks old, 20 ± 2 g) were housed under standard conditions, and mouse experiments were conducted with ethical approval from the Kunming Medical University Ethics Committee, adhering to Animal Research: Reporting of *In Vivo* Experiments (ARRIVE) guidelines. The mice were divided into phosphate-buffered saline (PBS) treated mice and EAE groups (n = 8 per group). EAE was induced via subcutaneous immunization with an antigen emulsion containing 1 mg of Myelin Oligodendrocyte Glycoprotein amino acids 35–55 (MOG35-55) peptide (Sigma-Aldrich) dissolved in 0.5 mL PBS and emulsified 1:3 (v/v) with complete Freund’s adjuvant (CFA, Sigma-Aldrich) using two glass syringes connected by a three-way stopcock for 2 hours. Each mouse received four subcutaneous injections (0.1 mL/site) at the inguinal region and three dorsal sites. Pertussis toxin (PTx, 200 ng per dose; Sigma-Aldrich) was administered intraperitoneally on days 0, 2, and 7 post-immunization to amplify neuroinflammation. Control mice received PBS injections following the same protocol. Disease progression was monitored daily using a clinical scoring system (0: no symptoms; 5: moribund state), and brain tissues were harvested at peak disease severity (14 days post-induction, EAE clinical scoring 3–4).

### Isolation of microglia

Microglia (CD11b+/CD45int) were isolated from brain tissues using a 30%/70% Percoll density gradient (800×g, 25 min, brake off) to enrich viable cells while minimizing myelin debris. After centrifugation, single-cell suspensions were stained with FITC-conjugated anti-CD11b antibody (clone M1/70, 1:200 dilution, Abcam Cat# ab24874) and PE-conjugated anti-CD45 antibody (clone EM-05, 1:100 dilution, Abcam Cat# ab269346) for 30 minutes at 4°C in the dark, followed by fluorescence-activated cell sorting (FACS) on a Beckman Coulter Moflo Astrios EQs (100 μm nozzle, 25 psi). The gating strategy (illustrated **Supplementary Figure S1**) excluded debris (FSC-A/SSC-A plot), dead cells (propidium iodide-negative, >95.81% viability), and non-microglial populations, retaining CD11b+/CD45int microglia (6.69% of total live cells). Post-sort reanalysis confirmed >99% purity (CD11b+/CD45int re-gating) and high sample integrity (RNA quality number >8.5, Agilent Bioanalyzer).

### RNA sequencing and analysis

**Figure 1:**
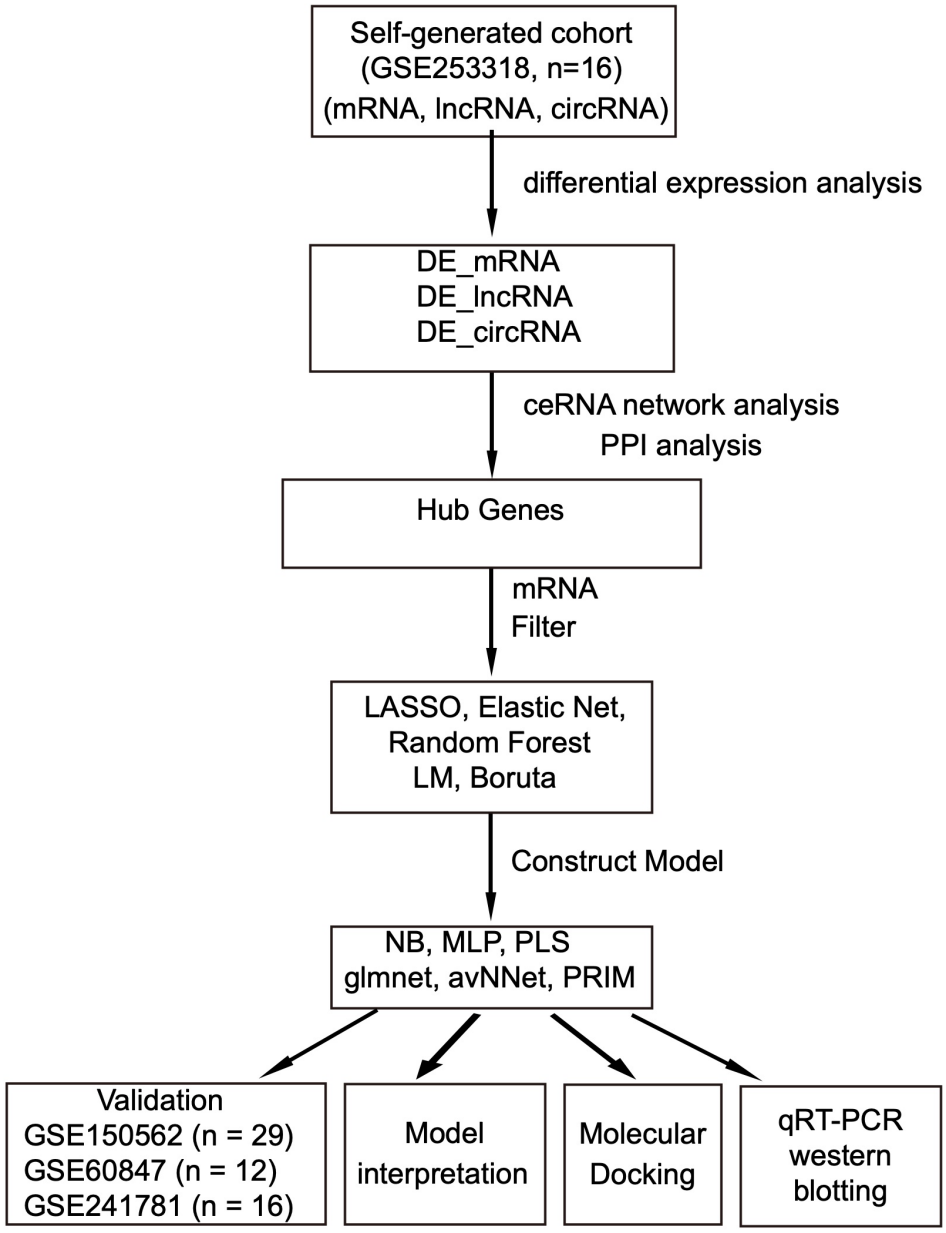
Flowchart of the research process. Data sources: self-generated RNA-seq, EAE mice brain tissue (GSE253318) and public microarray datasets: EAE mice spinal cord-GSE150562; EAE mice spinal cord-GSE60847; EAE & cuprizone mice brain-GSE241781. DE, differential expression; LASSO, least absolute shrinkage and selection operator; NB, naive Bayes; MLP, multilayer perceptron; avNNet, model-averaged neural network; PLS, partial least squares; PRIM, patient rule induction method; PPI, protein–protein interaction.

After establishing the animal model, we used bioinformatics approaches to identify hub genes and investigate the microglia-EAE relationship. Flowchart of the study was illustrated in **Figure 1**. RNA sequencing (RNA-seq) was performed on 16 microglia samples (8 EAE and 8 controls). Stranded RNA-seq libraries were sequenced on the Illumina NovaSeq 6000 platform using a 150 bp paired-end (PE150) configuration, generating 70–80 million raw reads per sample. Raw data were processed through stringent quality control: (1) removal of low-quality reads (reads with >50% bases having Phred score <20), (2) trimming of adapter sequences, (3) elimination of reads containing >5% ambiguous ‘N’ bases, and (4) filtering of host genome-derived contaminants by alignment to GRCm38. After filtering, clean data with Q30 ≥90% were retained for downstream analysis. Data processing included demultiplexing (bcl2fastq), alignment (HISAT2), and differential expression analysis (DESeq2). The resulting RNA-seq data were deposited in the GEO database for further accessibility (https://www.ncbi.nlm.nih.gov/geo/) under accession number GSE253318.

### Identification of differentially expressed mRNAs, lncRNAs and circRNAs in the microglia of EAE mice

To explore the potential mechanisms and biological significance of differentially expressed genes (DEGs) in EAE mice, we first used the edgeR package (23) to analyze the DEmRNAs, DElncRNAs and DEcircRNAs as illustrated in the study flowchart (**Supplementary Figure S2**). Differentially expressed circRNAs (DEcircRNAs), lncRNAs (DElncRNAs), microRNAs (DEmiRNAs), and mRNAs (DEmRNAs) were identified using a unified threshold of False Discovery Rate (FDR) < 0.05 and |log2FoldChange| > 1. Genes meeting these criteria were defined as significantly upregulated or downregulated.

The ggplot2 package (version 3.4.2) was used to visualize the results by constructing heatmaps, and the pheatmap package (version 1.0.12) was used to generate volcano plots. The expression levels of mRNAs and lncRNAs in each sample are presented as fragments per kilobase of transcript per million mapped reads (FPKM) values and the quantity of circRNAs in each sample is presented as reads per billion mapped reads (RPB) values as detailed in **Supplementary Tables S1**-**S3**.

### Construction of competing endogenous RNA networks

The predicted interactions between the screened DElncRNAs and miRNAs, DEcircRNAs and miRNAs, and miRNAs and DEmRNAs were predicted using the miRcode database (http://www.mircode.org/), the miRanda database (http://mirtoolsgallery.tech/mirtoolsgallery/node/1055) and the miRwalk database (24) (http://mirwalk.umm.uni-heidelberg.de/), respectively. These regulatory relationships were further validated using TargetScan, miRDB (25) and miRTarBase (26). Only interactions predicted by multiple databases were included. The combined miRNA-DElncRNA, miRNA-DEcircRNA and miRNA-DEmRNA interactions were used to construct the ceRNA network. The network diagram was generated using the ggplot2 package and Cytoscape.

To analyze posttranscriptional relationships between key genes and miRNAs, we identified miRNAs associated with the key genes through the miRNet database (27). An mRNA–miRNA regulatory network was then constructed and visualized with Cytoscape.

### Functional and pathway enrichment analysis of the DEGs

To investigate the functions and pathways enriched in the DEGs, we performed Gene Ontology (GO) analysis covering all three categories, including biological process (BP), molecular function (MF), and cellular component (CC), as well as Kyoto Encyclopedia of Genes and Genomes (KEGG) pathway enrichment analyses using the clusterProfiler R package (version 4.8.3) (28). The background gene set included all protein-coding genes annotated in the mouse genome (Ensembl GRCm38). Enrichment significance was adjusted for multiple testing using the Benjamini-Hochberg method (FDR < 0.05), while DEGs were identified with FDR-corrected q-values provided by OE Biotech Co., Ltd.

### Protein–protein interaction network analysis of the DEGs

We investigated the interactions between the DEmRNAs involved in the ceRNA network and a variety of factors, including transcription factors (TFs), miRNAs, small molecule drugs, and RNA binding proteins (RBPs). The PPI network of the DEGs was assembled utilizing the Search Tool for the Retrieval of Interacting Genes/Proteins (STRING) database (https://www.string-db.org/) (29). We subsequently exported the PPI data from the Search Tool for the Retrieval of Interacting Genes/Proteins (STRING) database using the parameters 0.4, 0.7, and 0.9 followed by visualization using Cytoscape (30). Furthermore, by employing the CytoHubba plugin (31), we further identified the critical subnetwork and hub genes within the PPI network.

### Identification of the DEGs via machine learning

To identify consensus DEGs with high accuracy and stability, we integrated five ML algorithms, each contributing unique strengths to the analysis. Elastic Net (a linear regression method combining L1 and L2 regularization) and Least Absolute Shrinkage and Selection Operator (LASSO) regression (32) [implemented via the glmnet package (33)] were used for feature selection. Elastic Net addressed multicollinearity by balancing L1 and L2 penalties, while LASSO applied L1 regularization to eliminate redundant predictors (34). Random Forest (RF), an ensemble tree-based method, ranked gene importance through bootstrap aggregation and entropy reduction, capturing non-linear relationships between genes and EAE phenotypes (35). To improve feature selection robustness, the Boruta algorithm, a wrapper method based on RF, iteratively compared original gene features to permuted “shadow” features, retaining only genes consistently outperforming noise (36). Linear regression served as a baseline model to identify genes with linear associations to disease status.

Hyperparameter optimization used stratified fivefold cross-validation with grid search. The dataset was stratified into five class-balanced folds, iteratively training on four folds and validating on one. Elastic Net (α: 0.1–0.9; λ: 10^−4^–10²) and LASSO (λ: 10^−4^-10²) were tuned via glmnet (37). LASSO results were visualized through cross-validation error curves and coefficient trajectory plots. Random Forest optimized mtry (√p, p/3, p/2) and tree count (500–2000) (34), while Boruta used default settings (36). Linear regression served as a baseline. The process was repeated 10 times with random seeds to ensure stability, and consensus DEGs were derived from consistent feature rankings.

Genes consistently selected by all five algorithms were aggregated into a final multigene signature. This signature was used to train predictive models for EAE classification and outcome prediction.

### Public data collection and processing

We evaluated three EAE gene expression datasets from the GEO database (https://www.ncbi.nlm.nih.gov/geo/) (38): GSE150562 (n = 29) (39), comprising microarray datasets from 24 EAE mice and 5 PBS-treated mice; GSE60847 (n = 12) (40), including 6 EAE and 6 PBS-treated mice; and GSE241781 (n = 16) (41), with samples from 6 EAE mice, 6 cuprizone-fed MS model mice, and 4 PBS-treated mice. All datasets contained CD11b+ microglia transcriptomes. Raw data were retrieved using the GEOquery R package (v2.68.0) (42) and preprocessed through quality control (probe filtering), normalization (quantile/log2), and batch correction. Probes with > 20% missing values were removed. For the Affymetrix MoGene-2.0-ST arrays (GPL16570; GSE150562 and GSE241781), quantile normalization was applied, while the Illumina MouseWG-6 v2.0 beadchip (GPL17543; GSE60847) data underwent log2 transformation. Batch effects in GSE241781 were mitigated using the ComBat algorithm (R sva package). Processed datasets were retained for downstream analysis (metadata in **Supplementary Table S4**).

Gene sets were obtained from the Molecular Signatures Database (MSigDB; https://www.gsea-msigdb.org/gsea/msigdb/) (43). The c5.go.v2023.2.Hs.symbols.gmt file provided GO terms, and the c2.cp.kegg.v7.4.symbols.gmt file supplied KEGG pathway gene sets for subsequent functional enrichment analysis.

### Screening and verification of biomarkers distinguishing healthy mice and mice with EAE

The EAE response classification model was trained with the multigene signature using six ML algorithms: multilayer perceptron (MLP), naive Bayes (NB), partial least squares (PLS), linear regression (glmnet), a model-averaged neural network (avNNet), and a patient rule induction method (PRIM). This diverse selection of models ensures that both linear and nonlinear patterns in the data are effectively captured while maintaining a balance between predictive accuracy and interpretability. For each ML algorithm with parameters, we use fivefold cross-validation (CV) to adjust the hyperparameters to optimize the performance of the model. To ensure robustness, we repeated the optimization process 10 times using different random seeds for each individual resampling.

The classifier, comprising a multigene signature derived from the aforementioned algorithmic model, was analyzed using validation datasets. The algorithm demonstrating the best classification efficacy in the validation set was selected to construct the EAE predictive model.

### Shapley Additive exPlanations values and residual analysis

To evaluate the interpretability and stability of the ML models, we employed the DALEX (Descriptive mAchine Learning EXplanations) package (44, 45), leveraging SHAP values and residual analysis as complementary approaches. SHAP values were calculated to quantify the contribution of each DEmRNA to individual model predictions. SHAP values for each DEmRNA were aggregated to evaluate their collective impact on model prediction, prioritizing genes with consistent directional effects on predictions. Residuals, defined as the differences between predicted and observed values, were computed for each model to evaluate stability. The distributions of residuals were visualized using boxplots, and models with lower median absolute residuals were identified as more stable and robust. This dual approach provided valuable insights into both the reliability and interpretability of the ML models used in our study.

### Molecular docking of the 6 key DEGs

Owing to accessibility-related challenges in the collection of brain samples from MS patients, the EAE animal model is a critical tool in the exploration of pharmaceuticals for MS treatment. Six DEGs in microglia were further analyzed for potential interactions with MS medications. Drug-target information was retrieved from the DrugBank database, and Structure Data Format (SDF) files for the drugs were retrieved from PubChem (https://pubchem.ncbi.nlm.nih.gov/). Target protein structures (PDB files) corresponding to the six DEGs were acquired from the Protein Data Bank (PDB; https://www.rcsb.org/).

Molecular docking calculations were subsequently performed with AutoDock 4.2.6. The Q site, defined as a putative ligand-binding pocket near the catalytic domain of the target protein, was enclosed within a 60 × 60 × 60 Å grid box to define the docking search space. The Lamarckian genetic algorithm was applied with 50 iterations, and default parameters were used for van der Waals and electrostatic scaling. Binding affinities were evaluated by selecting the lowest energy conformation from the largest cluster of docking poses.

### qRT–PCR validation

RNA was extracted from 16 microglia samples (8 EAE and 8 controls) using TRIzol and assessed for concentration, purity, and integrity. cDNA synthesis was performed with the riboSCRIPT Starter Kit, followed by qRT-PCR using SYBR Green chemistry on an ABI 7500 system. The mouse primer sequences are shown in **Table 1**. The reactions were conducted in triplicate, and gene expression was analyzed using the 2-ΔΔCt method with GAPDH as the internal control. Statistical significance between EAE and control groups was determined using an unpaired Student’s t-test (data normality confirmed via Shapiro-Wilk test), with *p* < 0.05 considered significant.

**Table 1:** Primer sequences used in the study.

GENE Name	Accession No.	Primer Sequence (5’→3’)	Length(bp)	Company
β-actin(m)-F	NM_007393.5	CTGGAGAAGAGCTATGAG	141	Beijing Tsingke Biotech Co., Ltd.
β-actin(m)-R		GATGGAATTGAATGTAGTTTC		
NGP (m) -F	NM_008694.2	AACTAAGATATGAGGAGATT	124	Beijing Tsingke Biotech Co., Ltd.
NGP (m) -R		ATATTGGTAGCAGGATTC		
Hist1h2bj (m) -F	NM_178198.3	GTCTACAAGGTGCTGAAG	80	Beijing Tsingke Biotech Co., Ltd.
Hist1h2bj (m) -R		TTCACGAACGAGTTCATG		
CD180 (m) -F	NM_008533.2	ATGGATGACGAAGATATTAGT	75	Beijing Tsingke Biotech Co., Ltd.
CD180 (m) -R		CTGTAGGTTGATGCTCTC		
PBLD1 (m) -F	NM_001359529.1	TGAAGTTGAAGACTTGATA	77	Beijing Tsingke Biotech Co., Ltd.
PBLD1 (m) -R		TTCTGGTATCTGTAGAGTA		
F10 (m) -F	NM_001242368.1	TTATGAAGAGGTCCGTGAA	75	Beijing Tsingke Biotech Co., Ltd.
F10 (m) -R		TCGCCGTCTTTATATTTGG		
MBNL3 (m) -F	NM_001310515.1	CTGATAATTCTGTGACAATCTG	83	Beijing Tsingke Biotech Co., Ltd.
MBNL3 (m) -R		GGGAGGAGGATGAAAGTA		
m-mmu_circ_0001569_F1	mmu_circ_0001569 (CircBase)	CAACAAGCCTGCCAAGGTG	140	RIBOBIO
m-mmu_circ_0001569_R1		GGCAGCAAATTAGCTTCATGTCT	140	

### Western blot analysis

Total proteins were extracted from EAE microglia using RIPA lysis buffer and PMSF (Beyotime, Shanghai). Protein samples were separated via 10% polyacrylamide gel electrophoresis, transferred to PVDF membranes (Immobilon, Thermo Fisher), and blocked with 5% non-fat dry milk for one hour. Membranes were incubated overnight with primary antibodies at 4°C, followed by a one-hour incubation with secondary antibodies at room temperature. Membrane analysis was performed using the ChemiDoc XRS+ imaging system (Bio-Rad). Antibodies targeting NGP (ab232676, 1:1000), HIST1H2BJ (ab157425, 1:1000), PBLD1 (ab215299, 1:1000), MBNL3 (ab243124, 1:1000), CD180 (ab113874, 1:1000), and F10 (ab228544, 1:1000) were from Abcam; GAPDH (5174, 1:1000) antibody were from Cell Signaling Technology.

### Statistical analysis

All the data processing and statistical analyses were conducted with R 4.0.2 software. The significance of differences between two continuous variables was assessed via an independent Student’s t test or the Wilcoxon rank-sum test for comparisons of two groups. The chi-square test or Fisher’s exact test was used to evaluate the significance of differences between two groups of categorical variables. Receiver operating characteristic (ROC) curve analysis was performed with the pROC R package. All the statistical tests were two-tailed, and *p* < 0.05 was considered to indicate statistical significance. Western blot band intensities were quantified via ImageJ software (NIH) through densitometric analysis, with background subtraction and normalization to internal controls (GAPDH), using triplicate biological replicates.

## Results

### Analysis of the DEmRNAs, DElncRNAs and DEcircRNAs

We employed the edgeR algorithm to detect mRNAs, lncRNAs, and circRNAs exhibiting significant differential expression between microglia derived from EAE mice and those derived from PBS-treated mice. Specifically, we identified 141 significantly upregulated mRNAs and 27 significantly downregulated mRNAs (**Supplementary Figures S3A, D**; **Supplementary Table S5**) in EAE mice compared with the PBS-treated mice. Among the identified lncRNAs, 154 were significantly upregulated, and 193 were significantly downregulated (**Supplementary Figures S3B, E**; **Supplementary Table S6**). Moreover, we identified a total of 359 significantly upregulated circRNAs and 476 significantly downregulated circRNAs (**Supplementary Figures S3C, F**; **Supplementary Table S7**).

### Potential ceRNA networks and functional enrichment analysis

**Figure 2:**
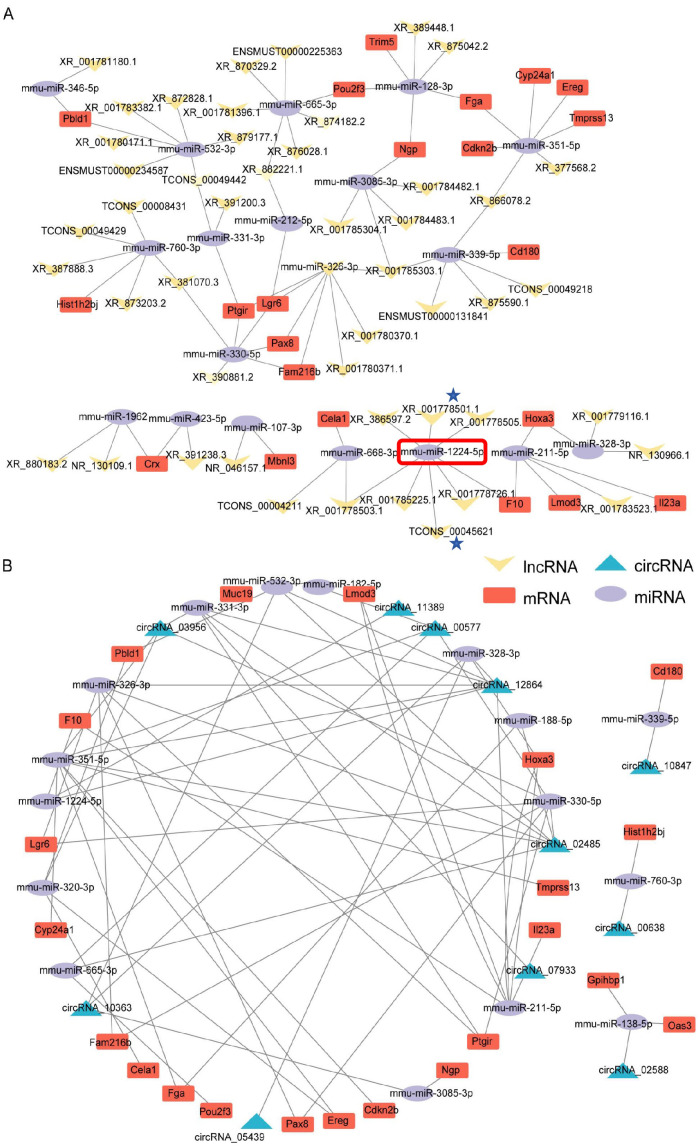
ceRNA network analysis of the self-generated RNA-seq dataset (EAE mice brain-GSE253318). **(A)** lncRNA–miRNA–mRNA ceRNA network interaction analysis. Key annotations: *mmu-miR-1224-5p* (highlighted in red) targets seven lncRNAs, including *XR_001778501–1* and *TCONS_00045621* (marked with asterisks). Node size reflects interaction degree; edges represent predicted miRNA-target binding. **(B)** circRNA–miRNA–mRNA ceRNA network interaction analysis.

We further examined the regulatory associations among the differentially expressed mRNAs, lncRNAs, and circRNAs. Our findings revealed the pivotal roles of miRNAs in the lncRNA–miRNA–mRNA ceRNA interaction network, revealing notable interconnectedness within the network (**Figure 2**). For example, the miRNA *mmu-miR-1224-5p* (highlighted in red in **Figure 2A**) was found to target and regulate seven lncRNAs, including *XR_001778501–1 and TCONS_00045621* (labeled with asterisks in **Figure 2A**). Additionally, within the circRNA–miRNA–mRNA ceRNA network, we observed significant interactions among the miRNA nodes (**Figure 2B**; **Supplementary Table S8**).

To determine the chromosomal positions of the DEmRNAs, DElncRNAs, and DEcircRNAs within the ceRNA network and to understand the relative spatial distribution of these interacting molecules, we initially analyzed the localization data obtained from ENCODE (file: Mus_musculus.GRCm39.110.gtf) for visualization using TBtools. Our findings revealed that the DElncRNAs located on chromosomes 8, 9, 11, and 14 were relatively more abundant than those located on other chromosomes (**Supplementary Figure S4**; **Supplementary Table S9**). Conversely, DEmRNAs and DEcircRNAs within the ceRNA network were dispersedly distributed across various chromosomes without evident clustering patterns (**Supplementary Figure S4**).

**Figure 3:**
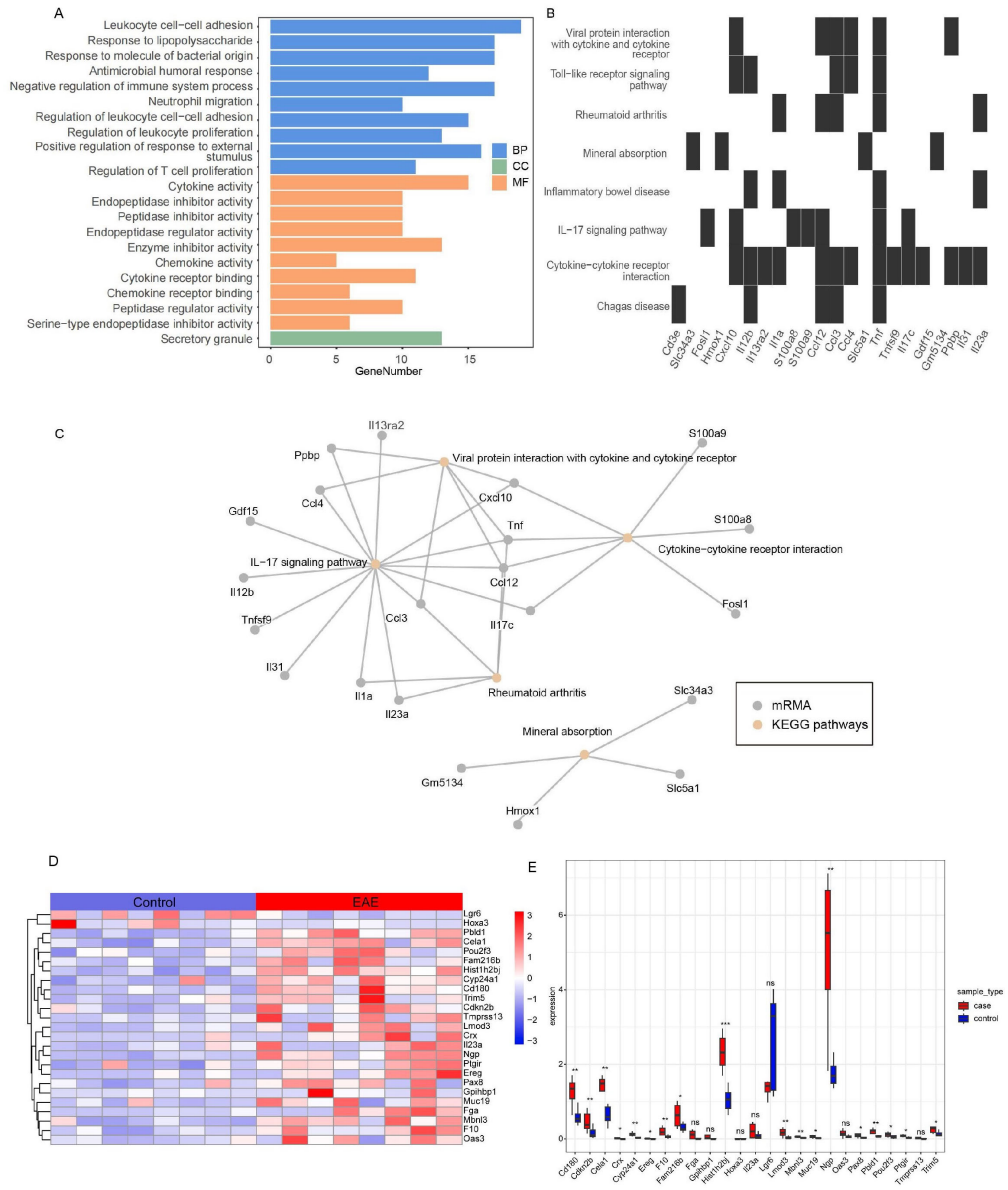
Integrated analysis of functional enrichment and key mRNA expression in the ceRNA network of self-generated RNA-seq dataset (EAE mice brain-GSE253318). **(A)** GO functional enrichment analysis (BP, CC, MF). **(B)** KEGG pathway enrichment analysis. **(C)** Network diagram of significantly enriched KEGG pathways and associated genes. **(D)** Heatmap of key mRNA expression in the ceRNA network (red: high expression; blue: low expression; values normalized to z-scores). **(E)** Box plot of differential expression of key mRNAs across experimental groups (ns, not significant; *FDR < 0.05, **FDR < 0.01, ***FDR < 0.001). GO, Gene Ontology; KEGG, Kyoto Encyclopedia of Genes and Genomes; BP, biological process; CC, cellular component; MF, molecular function.

GO analysis revealed significant enrichment of the DEmRNAs in the ceRNA network (FDR < 0.05) in 313 immune-related terms, including leukocyte adhesion, lipopolysaccharide response, and neutrophil migration (**Figure 3A**; **Supplementary Table S10**). KEGG analysis identified significant enrichment (FDR < 0.05) in immune pathways such as IL-17 signaling, cytokine receptor interactions, and Toll-like receptor (TLR) signaling (**Figure 3B**; **Supplementary Table S11**). Notably, these pathways shared gene nodes, suggesting that the DEmRNAs modulate various biological pathways in EAE progression (**Figure 3C**). Therefore, these DEmRNAs are likely to be involved in microglial activation and adhesion, positioning them as potential biomarkers and therapeutic targets for MS.

We examined the expression profiles of the DEmRNAs in the ceRNA network and found that most were upregulated in EAE mice, except for Leucine-rich repeat-containing G protein-coupled receptor 6 (*LGR6)* and Homeobox A3 (*HOXA3)* genes (**Figures 3D, E**). Further analysis of expression correlation among the differentially expressed mRNAs, lncRNAs, and circRNAs revealed distinct patterns. Notably, the expression trends of *LGR6* and *HOXA3* with DElncRNAs differed markedly from those of other DEmRNAs (**Supplementary Figure S5A**), a pattern also observed between DEmRNAs and DEcircRNAs (**Supplementary Figure S5B**). Correlation analysis of DEcircRNAs and DElncRNAs suggested potential underlying mechanisms (**Supplementary Figure S5C**).

### Interaction analysis of the EAE DEmRNAs involved in the ceRNA network for biological function prediction

We investigated the interactions among the DEmRNAs involved in the ceRNA network and a variety of factors, including TFs, miRNAs, small molecule drugs, and RBPs. The PPI network constructed for the DEmRNAs and TFs revealed interactions, particularly for Lymphocyte Antigen 180 (*CD180*) and Muscleblind-Like Protein 3 (*MBNL3*), which not only interact with each other but are also closely associated with TFs. Notably, the mRNAs *CD180* and Interleukin 23 Subunit Alpha (*IL23a*) frequently interacted with TFs (**Supplementary Figures S6A**, **S7**). The constructed mRNA–drug interaction network identified Phenazine Biosynthesis-Like Domain-Containing Protein 1 (*PBLD1*) and Neutrophilic Granule Protein (*NGP*) as potential drug targets (**Supplementary Figures S6B**, **S7**). Furthermore, the DEmRNA–RBP interaction network revealed that the mRNAs Coagulation Factor X (*F10*), Cyclin-Dependent Kinase Inhibitor 2B (*CDKN2B*), Fibrinogen Alpha Chain (*FGA*), Cone-Rod Homeobox (*CRX*), Epiregulin (*EREG*), *IL23a*, and *LGR6* may interact with several RBPs. (**Supplementary Figures S6C**, **S7**).

### Identification of EAE biomarkers and validation with 3 public datasets

A total of five ML algorithms, namely, the elastic net, LASSO, RF, Boruta, and linear regression algorithms, were applied to further identify key DEmRNAs. Nine significantly DEGs were identified by LASSO regression (**Supplementary Figure S8A**); elastic network analysis identified 10 genes (**Supplementary Figure S8B**); linear regression and the RF algorithm identified 25 genes each (**Supplementary Figures S8C, D**); and the Boruta algorithm identified 12 significant genes (**Supplementary Figure S8E**). As shown in the UpSet diagram (**Supplementary Figure S8F**), a total six genes were commonly identified by each method, namely, *NGP*, Histone Cluster 1 H2B Family Member J (*HIST1H2BJ*), *PBLD1, MBNL3*, *CD180*, and *F10*. All six genes were upregulated in the in-house dataset, with |log_2_FC| values of 1.56, 1.08, 1.44, 1.01, 1.20, and 1.63, respectively (*p* < 0.05) (**Supplementary Table S5**).

### Construction and validation of ML models for determination of the microglia-associated multigene signature

We employed six ML algorithms (NB, MLP, avNNet, PLS, PRIM, and glmnet) to assess the predictive performance of a six-DEmRNA signature for EAE. All models demonstrated high area under the curve (AUC) values (NB: 1, MLP: 1, avNNet: 1, PLS: 0.938, PRIM: 0.875, glmnet: 1) (**Supplementary Figure S8G**), along with favorable C-indexes and F1 scores (**Supplementary Figures S8H**; **Supplementary Table S12**), indicating robust predictive performance.

Validation in three independent EAE datasets further confirmed the accuracy of the model. For the GSE150562 dataset, PLS, MLP, and avNNet models achieved high AUC values (PLS: 0.892, MLP: 0.892, avNNet: 0.833) (**Supplementary Figure S8I**), with PLS and MLP models showing AUCs as high as 0.892 but with lower F1 scores (**Supplementary Figure S8J**), indicating stable performance. In the GSE60847 dataset, PLS (AUC = 0.833) and avNNet (AUC = 0.792) models displayed superior predictive efficacy (**Supplementary Figure S8K**), with higher C-indexes and F1 scores than other models (**Supplementary Figure S8L**).

For the GSE241781 dataset, the MLP (AUC = 0.917) and avNNet (AUC = 0.938) models showed optimal performance (**Supplementary Figure S8M**), with higher C-indexes and F1 scores than other algorithms (**Supplementary Figure S8N**). These results suggest that the avNNet model exhibited the most consistent predictive ability, while the PLS and MLP models were somewhat less stable.

### Interpretability and stability determined by SHAP values and residual analysis

We used the DALEX package to assess the explanatory capacity of each DEmRNA within the prediction models (**Supplementary Figures S8O-V**). The NB model had the lowest residual, with the median residual closely approximating 0, indicating its stability and robust predictive performance (**Supplementary Figures S8O, P**). The predictive efficacy of the six-gene signature was evaluated across the avNNet, PLS, and MLP models, which demonstrated high prediction scores (0.721, 0.815, and 0.811, respectively). SHAP values were calculated to determine the contribution of each DEmRNA, with *NGP* consistently showing the highest contribution across all models (7.122 for avNNet, PLS, and MLP) (**Supplementary Figures S8Q-V**).

The combination of residual and SHAP value analyses confirmed the robustness, stability, and interpretability of the models. Residual analysis validated the stability, particularly for the NB model, while the SHAP values highlighted the biological relevance of individual biomarkers. These results affirm the consistency and interpretability of the predictions of the avNNet, PLS, and MLP models, reinforcing the predictive power of the six-gene signature and identifying *NGP* as a key contributor to the model predictions.

### Molecular docking of the 6 key microglia-associated DEGs

**Figure 4:**
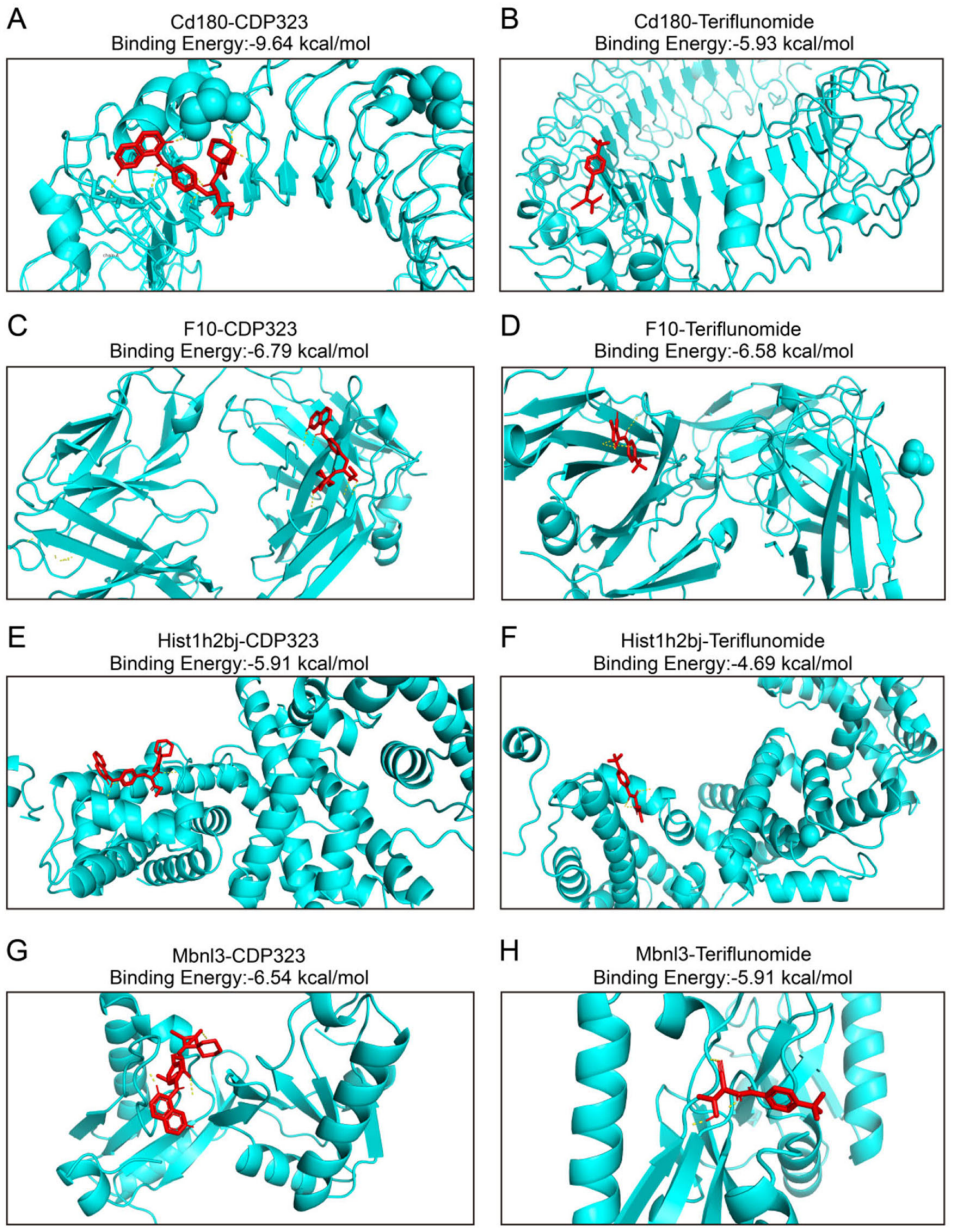
Molecular docking of the proteins encoded by CD180, F10, HIST1H2BJ, and MBNL3 with CDP323 and teriflunomide. Molecular docking and binding energies for **(A)** CD180 and CDP323, **(B)** CD180 and teriflunomide, **(C)** F10 and CDP323, **(D)** F10 and teriflunomide, **(E)** HIST1H2BJ and CDP323, **(F)** HIST1H2BJ and teriflunomide, **(G)** MBNL3 and CDP323, and **(H)** MBNL3 and teriflunomide.

Molecular docking was conducted to identify potential interactions between MS drugs and the six DEGs. Data from DrugBank indicated that teriflunomide and CDP323 interacted with four of the DEGs. Docking results showed that CD180 had binding energies of -9.64 kcal/mol with CDP323 and -5.93 kcal/mol with teriflunomide (**Figures 4A, B**). F10 exhibited binding energies of -6.79 kcal/mol with CDP323 and -6.58 kcal/mol with teriflunomide (**Figures 4C, D**). HIST1H2BJ and MBNL3 showed binding energies of -5.91 kcal/mol and -4.69 kcal/mol, respectively, with CDP323 and -6.54 kcal/mol and -5.91 kcal/mol with teriflunomide (**Figures 4E–H**). Notably, no significant binding (binding energy ≥ -5 kcal/mol) was observed between the MS drugs and the remaining two biomarkers, NGP and PBLD1.Binding energies below -5 kcal/mol are typically considered significant for docking results. Thus, the favorable binding affinities observed for CD180, F10, HIST1H2BJ, and MBNL3 with CDP323 and teriflunomide suggest that these proteins could serve as potential therapeutic targets for MS treatment.

### Verification of the microglia-associated markers

**Figure 5:**
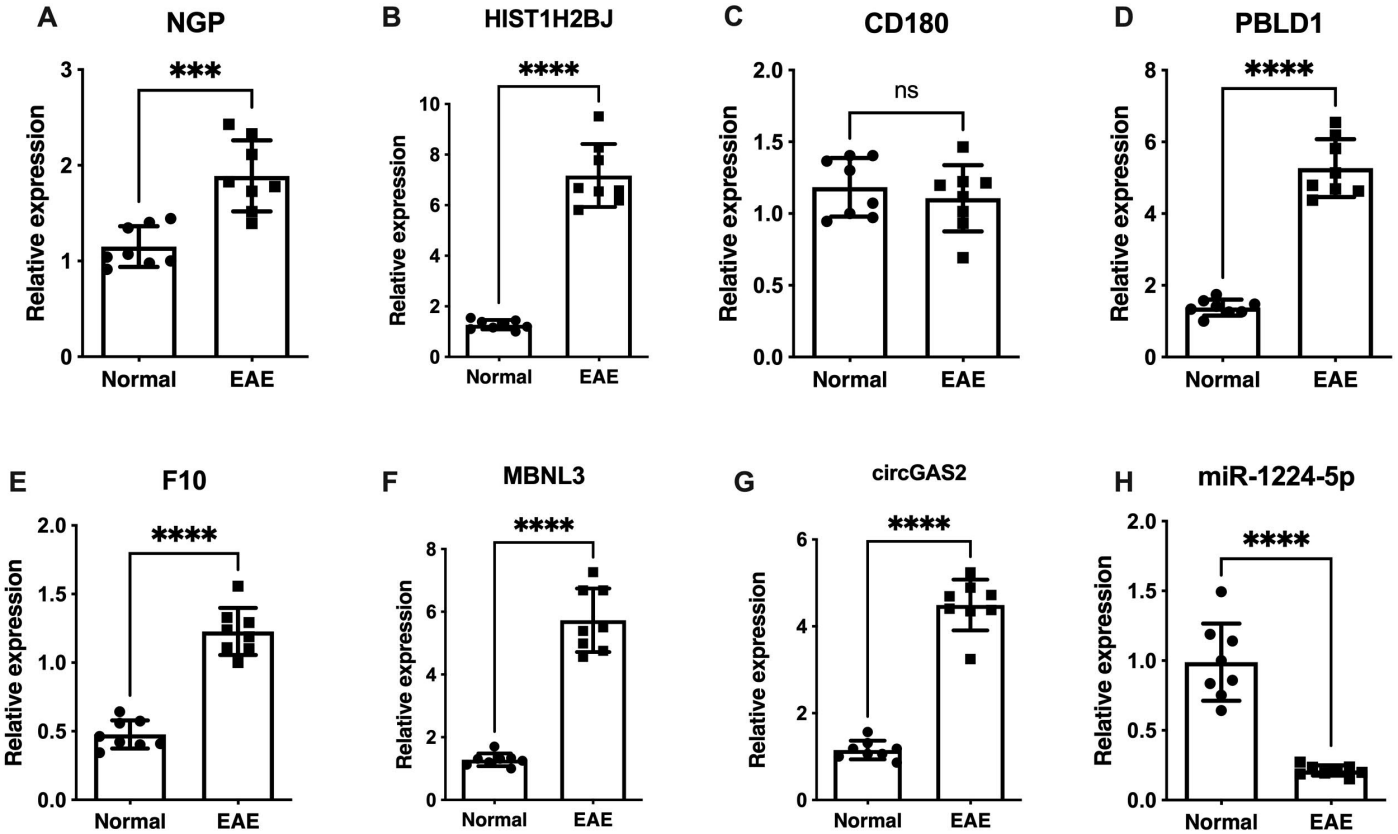
Verification of *NGP, CD180, HIST1H2BJ, PBLD1, MBNL3, F10, mmu-circ-0001569* and *miR-1224-5p* expression by qRT–PCR. **(A)** *NGP*, **(C)** *HIST1H2BJ*, **(D)** *MBNL3*, **(E)** *PBLD1*, and **(G)** *mmu-circ-0001569* were significantly upregulated in EAE microglia compared with control microglia (*p* < 0.001). **(F)** *F10* and **(H)** *miR-1224-5p* were significantly downregulated in EAE microglia (*p* < 0.001). **(B)** *CD180* expression did not significantly differ between EAE microglia and control microglia. ns: not statistically significant; ***p < 0.001, ****p < 0.0001.

**Figure 5** shows the expression levels of six biomarkers in 16 tissue samples (8 normal samples and 8 EAE samples) measured by qRT–PCR. The fold changes in various transcript levels were determined using the ΔΔCT methodology (**Supplementary Table S13**). *NGP, HIST1H2BJ, PBLD1, MBNL3* and a DEcircRNA of interest (*mmu-circ-0001569*) were significantly upregulated in EAE microglia (*p* < 0.001), and *F10* and *miR-1224-5p* were significantly downregulated (*p* < 0.001) (**Figure 5**), indicating that the results were reproducible and reliable. Western blot analysis of the relative protein expression levels of NGP, HIST1H2BJ, PBLD1, MBNL3, CD180, and F10 correlated well with the qRT–PCR results, demonstrating significant upregulation in all targets except CD180 (**Supplementary Figures S9**, **S10**; **Supplementary Table S14**).

## Discussion

Microglia play pivotal roles in EAE and MS progression by driving immune activation, antigen presentation, and myelin phagocytosis (6, 7). Emerging evidence highlights that microglia modulate neuroinflammation not only through direct cell-cell interactions but also via the secretion of cytokines (e.g., IL-1β, TNF-α), chemokines (e.g., CCL2, CXCL10), and ROS (1, 2, 5, 6, 12), which amplify adaptive immune responses and exacerbate demyelination. Our focus on post-transcriptional mechanisms stems from their critical role in fine-tuning these inflammatory outputs, as non-coding RNAs and RNA-binding proteins regulate the stability and translation of mRNAs encoding immune mediators (46, 47).

This study revealed significant changes in microglial gene expression and leveraged ceRNA and PPI networks to identify posttranscriptional regulatory mechanisms. ceRNA networks (lncRNA/circRNA–miRNA–mRNA) modulate microglial inflammation in EAE by competitively sequestering miRNAs from target mRNAs via shared miRNA response elements, thereby derepressing inflammatory transcripts (48–50). The six identified DEmRNAs integrate into these lncRNA/circRNA–miRNA–mRNA axes, suggesting their roles as either effectors or modulators of ceRNA-driven immune dysregulation. Among the identified circRNAs, mmu-circ-0001569 (circGAS2) showed significant upregulation in EAE microglia. This circRNA has not been previously reported in neuroinflammatory models, suggesting its potential microglia-specific roles warranting further investigation.

The chromosomal distribution patterns of ceRNA network components in EAE microglia may reflect both structural and functional genomic organization. The enrichment of DElncRNAs on chromosomes 8, 9, 11, and 14 aligns with known immune-related genomic hotspots in mice. Chromosome 11 harbors the innate immunity cluster (51, 52), while chromosome 14 contains genes correlated with several neurological diseases [e.g., Alzheimer’s disease (53) and epilepsy (54)]. In contrast, the dispersed distribution of DEmRNAs and DEcircRNAs across chromosomes implies system-wide regulatory integration rather than locus-specific control.

RNA sequencing, including bulk and single-cell approaches, has been used extensively to study the functions of microglia in MS and EAE (3, 47). However, discrepancies in gene expression profiles between studies stem from methodological variations and model-specific differences (55, 56). Using bioinformatics and ML methods, we identified six novel biomarkers (*NGP, HIST1H2BJ, PBLD1, MBNL3, CD180*, and *F10*) that were up-regulated in EAE microglia, none previously linked to EAE. Validation across datasets confirmed consistent expression patterns and high diagnostic efficacy. Among these biomarkers, *NGP, HIST1H2BJ*, and *CD180* contributed most to the differences between EAE and healthy microglia. Interaction network analyses revealed their integration in ceRNA networks, with *NGP*, *MBNL3*, and *CD180* interacting with TFs, underscoring their key regulatory roles in the EAE immune microenvironment.

NGP, a cystatin family member, modulates innate and adaptive immunity by attenuating TLR4 signaling and enhancing phagocytosis by macrophages and microglia (57). Given that TLR4 promotes the activity of proinflammatory cytokines such as TNF-α and IL-1β, which exacerbate MS inflammation, our finding that NGP is upregulated suggests that it may be involved in TLR4-associated pathways (57). Similarly, CD180, another TLR family member, modulates immune responses in B cells and dendritic cells through NF-κB and MAPK signaling (58, 59). Dysregulation of CD180 expression is linked to MS pathogenesis (60, 61), but no significant change in CD180 expression were observed in our qRT-PCR and Western blot experiments. The observed discrepancy may arise from the higher sensitivity of sequencing in detecting subtle transcriptional changes, combined with technical variability in qRT-PCR (e.g., primer efficiency) and Western blot (e.g., antibody specificity), or biological factors such as post-transcriptional regulation uncoupling mRNA and protein expression levels. Further investigation using single-cell RNA sequencing and spatial transcriptomics is necessary to explore CD180 expression in microglial subtypes, along with functional studies to clarify the roles of NGP and CD180 in TLR-mediated immune regulation in MS.

*HIST1H2BJ*, a histone gene involved in nucleosome structure and immune defense, shows elevated expression in autoimmune disorders such as rheumatoid arthritis (62, 63). In this study, increased *HIST1H2BJ* expression in EAE microglia suggests impaired phagocytosis and prolonged exposure of apoptotic cells to the immune system (61), likely contributing to immune dysregulation in MS.

*PBLD1*, a tumor suppressor gene that is upregulated in EAE microglia, attenuates TNF-α-induced inflammation by suppressing NF-κB activity (64, 65), indicating the activation of anti-inflammatory pathways. This upregulation likely represents a compensatory response to counterbalance excessive neuroinflammation in acute EAE. MBNL3, an RNA-binding protein involved in RNA metabolism and splicing, has been linked to diseases such as autism and amyotrophic lateral sclerosis (66, 67). In the EAE context, MBNL3 may mediate immune-metabolic crosstalk by fine-tuning post-transcriptional regulation of inflammatory mediators, though its precise role in MS remains undefined. While our findings implicate PBLD1 and MBNL3 in EAE-associated immune regulation, their pathophysiological significance in human MS requires validation through microglia-specific knockout models and analysis of MS patient datasets.

The six biomarkers participate in key pathways (e.g., LPS response, TLR/IL-17 signaling) through multilayered regulatory networks. PPI analysis revealed their interactions with TFs, RBPs, and small-molecule drugs: (1) *CD180* and *MBNL3* associate with TFs (JUN, ETS1, IRF4, etc.), forming a pro-inflammatory feedforward loop via the CD180/IL23a axis (68–74); (2) *F10* and *CD180* bind RBPs (e.g., IL23R, IL12RB1) (75), implicating post-transcriptional control in cytokine-receptor pathways; while (3) *PBLD1* and *NGP* interact with therapeutic compounds, suggesting druggable potential. These TF/RBP networks collectively establish a synergistic framework for microglial dysregulation in EAE and MS pathogenesis.

**Figure 6:**
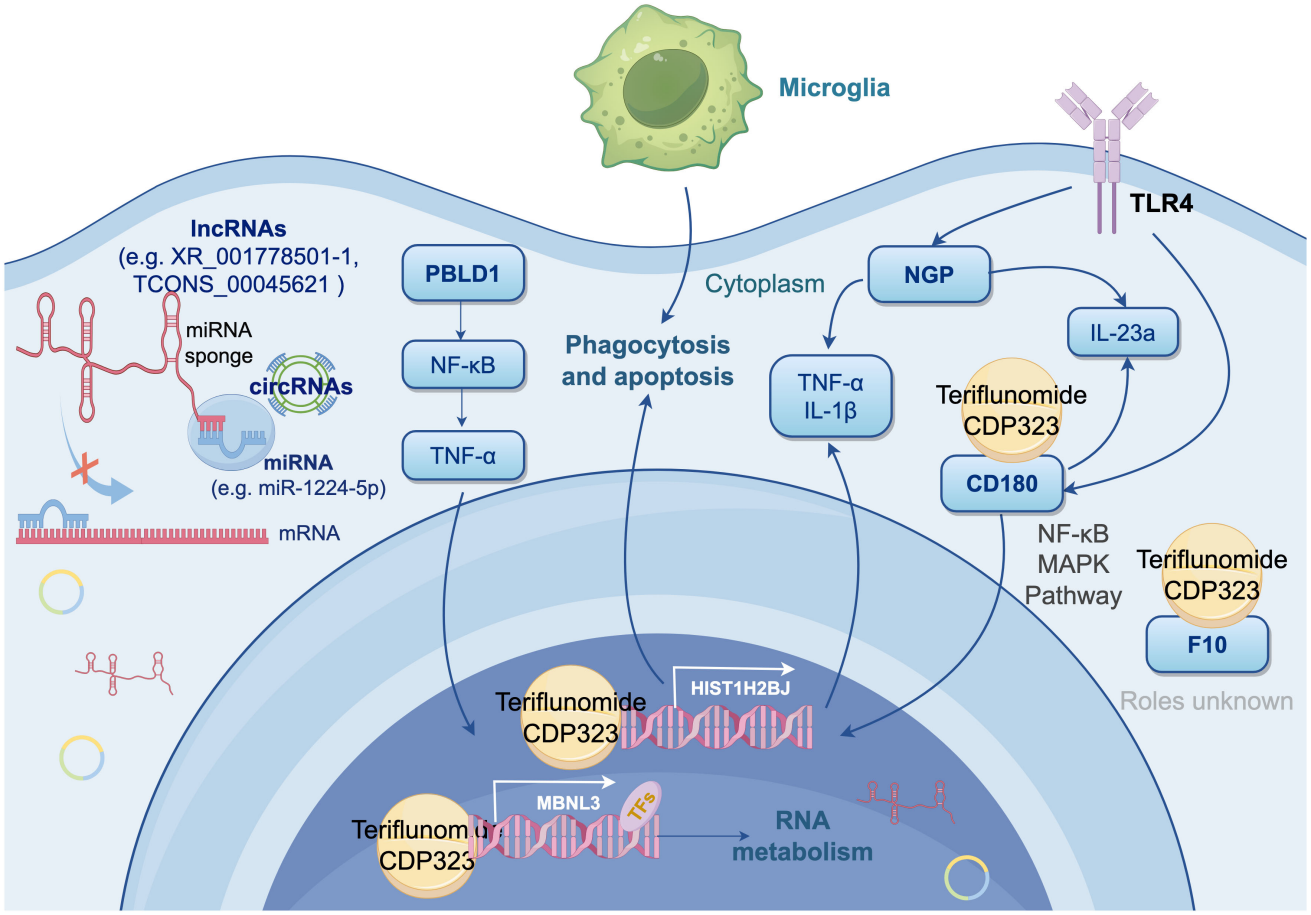
Integrated Mechanistic Model of Signature Genes in Microglial Activation and Neuroinflammation (by Figdraw 2.0). This diagram proposes a synergistic network involving six DEmRNAs (*NGP, HIST1H2BJ, PBLD, MBNL3, CD180*, and *F10*) through multiple mechanism, driving microglial activation and neuroinflammation. Key elements include: NGP suppresses TLR4 to attenuate neuroinflammation; HIST1H2BJ impairs apoptotic clearance and phagocytosis, exacerbating autoimmunity; PBLD antagonizes NF-κB/TNF-α signaling; MBNL3 fine-tunes RNA splicing in immunometabolism; CD180 modulates B-cell-like responses via NF-κB/MAPK, cytoplasmic ceRNA crosstalk (e.g., lncRNAs XR_001778501.1/TCONS_00045621 sponging miR-1224-5p), and TF-mediated regulation of *MBNL3*. Molecular docking highlights interactions between signature proteins and MS drugs (teriflunomide, CDP323), suggesting therapeutic targets.

Microglial heterogeneity is well-characterized by canonical markers: TMEM119 and P2RY12 distinguish resident microglia from macrophages, while TREM2 marks activated states in EAE and MS (76, 77). Our newly identified biomarkers (*NGP, HIST1H2BJ, CD180, PBLD1, MBNL3, F10*) show no overlap with these traditional markers, indicating distinct state-dependent transcriptional programs in CD11b+/CD45int subtype of microglia of acute EAE. Importantly, none exhibit vascular endothelial expression in neuroinflammatory contexts (Human Protein Atlas/GEO/Brain RNA-seq), supporting microglia-specific roles. These interactions are synthesized in **Figure 6** as a mechanistic framework for MS therapeutic targeting.

Molecular docking revealed interactions between four candidate proteins (CD180, F10, HIST1H2BJ, MBNL3) and MS therapeutics: CD180-teriflunomide binding (-5 kcal/mol) implicates TLR4 modulation, while F10-CDP323 interaction suggests coagulation pathway involvement. HIST1H2BJ and MBNL3 show potential for epigenetic/splicing-targeted therapies. Although NGP and PBLD1 lacked drug affinity, their established roles in environmental response (NGP in neurotoxin clearance, PBLD1 in detoxification) support their selection as targets for MS’s environmental-inflammatory axis.

While our models demonstrated robust performance in the independent validation set, potential overfitting risks may arise from the moderate sample size and tissue-specific transcriptomic biases. External validation across diverse populations (e.g., progressive MS subtypes) and omics modalities (e.g., proteomics) for further researches is essential. While molecular docking predicts interactions between CD180/F10/HIST1H2BJ/MBNL3 and MS drugs (binding energy < -5 kcal/mol), experimental validation through cryogenic electron microscopy and functional assays remains essential for confirmation.

To bridge our findings to clinical translation, future studies will prioritize validation of *NGP, HIST1H2BJ, CD180, PBLD1, MBNL3*, and *F10* in longitudinal EAE models and human MS lesion to assess their temporal dynamics during disease progression and remission. For functional validation, gene-edited mice could be used to test the causal roles of microglial drug targets in neurovascular dysfunction and clarify the therapeutic relevance of these biomarkers and accelerate their translation into precision immunomodulatory strategies for MS.

## Conclusions

In this study, *NGP, HIST1H2BJ, PBLD1, MBNL3, CD180*, and *F10* were identified as novel microglial biomarkers for EAE, verified in GEO datasets, and shown to interact with MS-related molecules and medications. These findings provide a reference for MS diagnosis and treatment, highlighting these six genes as potential targets for immunoregulatory interventions.

## Data Availability

The datasets presented in this study can be found in online repositories. The names of the repository/repositories and accession number(s) can be found in the article/**Supplementary Material**.

## Supplementary material

Supplementary Table 1 FPKM values of the mRNAs in each sample for self-generated RNA-seq dataset (EAE mice brain-GSE253318).

Supplementary Table 2 FPKM values of the lncRNAs in each sample for self-generated RNA-seq dataset (EAE mice brain-GSE253318).

Supplementary Table 3 RPB (junction reads per billion mapped reads) of the circRNAs in each sample for self-generated RNA-seq dataset (EAE mice brain-GSE253318).

Supplementary Table 4 GEO datasets used in this study.

Supplementary Table 5 Exprssion of DEmRNAs (log_2_|FC| > 1, FDR < 0.05) in self-generated RNA-seq dataset (EAE mice brain-GSE253318).

Supplementary Table 6 Expression of DElncRNAs (log_2_|FC| > 1, FDR < 0.05) in self-generated RNA-seq dataset (EAE mice brain-GSE253318).

Supplementary Table 7 Expression of DEcircRNAs (log_2_|FC| > 1, FDR < 0.05) in self-generated RNA-seq dataset (EAE mice brain-GSE253318).

Supplementary Table 8 Average node degrees of different RNAs in the circRNA–miRNA–mRNA ceRNA network of self-generated RNA-seq dataset (EAE mice brain-GSE253318).

Supplementary Table 9 Details on the DElncRNAs located on chromosomes 8, 9, 11, and 14 in self-generated RNA-seq dataset (EAE mice brain-GSE253318).

Supplementary Table 10 GO terms enriched in the DEmRNAs involved in the ceRNA network of self-generated RNA-seq dataset (EAE mice brain-GSE253318).

Supplementary Table 11 KEGG pathways enriched in the DEmRNAs involved in the ceRNA network of self-generated RNA-seq dataset (EAE mice brain-GSE253318).

Supplementary Table 12 Model performance of the 6 ML algorithms on the self-generated RNA-seq dataset (EAE mice brain-GSE253318) and EAE mice spinal cord-GSE150562, EAE mice spinal cord-GSE60847, and EAE & cuprizone mice brain-GSE241781 datasets.

Supplementary Table 13 Original 2-△△Ct values of qRT-PCR.

Supplementary Table 14 Densitometric analysis of Western blot bands for NGP, HIST1H2BJ, MBNL3, CD180, and F10, normalized to GAPDH expression levels (mean ± SEM, n=3; **p <*0.05, ***p* < 0.01, ****p <* 0.001, two-way ANOVA*).*

Supplementary Table 15 A complete alphabetical list of abbreviations of key terms, genes, and pathways in the study.
