# Supplementary material for: Identification of the microglia-associated signature in experimental autoimmune encephalomyelitis
Source: Front Immunol. 2025 Jun 5;16:1581878. doi: 10.3389/fimmu.2025.1581878 (PMC12176564; doi:10.3389/fimmu.2025.1581878)
Supplement: Supplementary Table 15 — A complete alphabetical list of abbreviations of key terms, genes, and pathways in the study. [file SupplementaryFile1.pdf]

Supplementary Material

Supplementary Figures

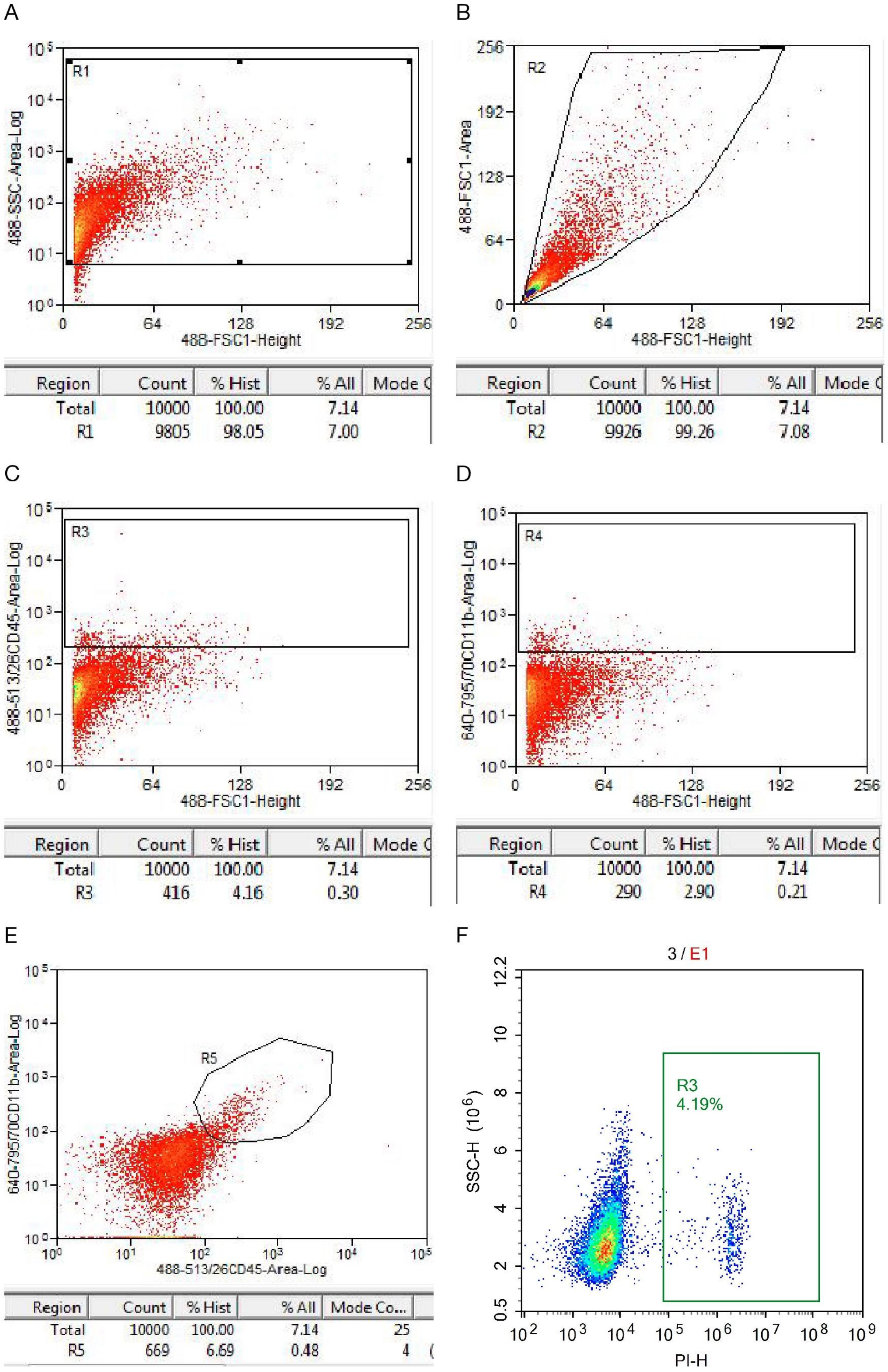

Figure S1. Flow Cytometry Gating Strategy for Microglial Isolation

(A) Debris exclusion: Initial events were gated on a FSC-A (forward scatter area) vs. SSC-A (side scatter area) plot to exclude small debris (lower left quadrant). Intact cells (R1: 98.05% of total events) were retained.

(B) Single-cell selection: FSC-H (height) vs. FSC-W (width) plot applied to R1 to exclude doublets and aggregates, retaining single cells (R2: 99.26% of R1).

(C) CD45 expression profiling: CD45-FITC (FL1) vs. SSC-A plot gated on R2. CD45+ cells (R3: 4.16% of R2) were selected, excluding CD45− populations.

(D) CD11b expression profiling: CD11b-APC-Cy7 (FL4) vs. SSC-A plot gated on R3. CD11b+ cells (R4: 2.9% of R2) were isolated.

(E) Microglial identification: CD45 (x-axis) vs. CD11b (y-axis) dual-parameter plot gated on R3. CD11b+/CD45int microglia (R5: 6.69% of R3) were sorted (upper right circle).

(F) Propidium iodide (PI) staining (5 μg/mL, 15 min) confirmed 95.81% viability (PI-negative cells).

Gating hierarchy: R1 → R2 → R3 → R4 → R5.

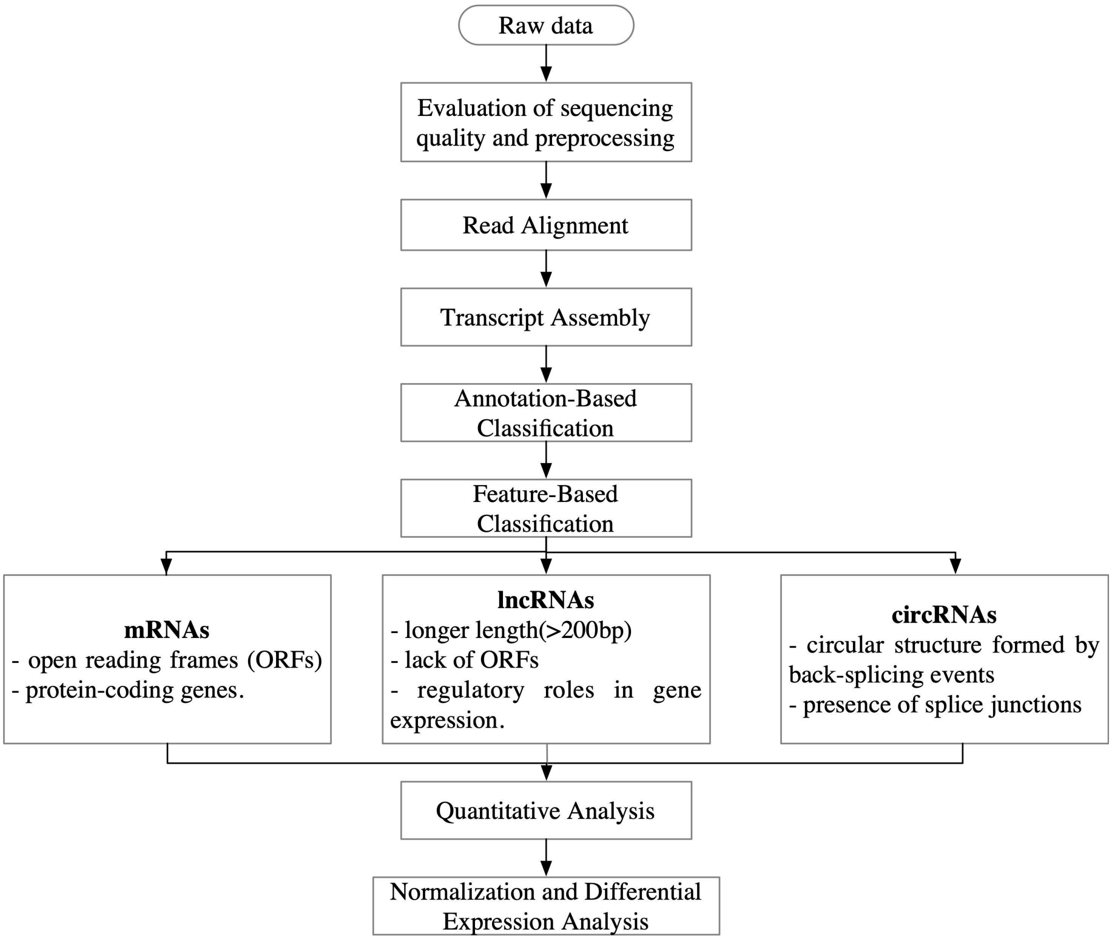

Figure S2. Flowchart of the RNA sequencing analysis and classification of the mRNAs, lncRNAs, and circRNAs in self-generated RNA-seq dataset (EAE mice brain-GSE253318).

Read alignment: The initial sequencing data were processed by alignment with a reference genome or transcriptome with HISAT2, which facilitates mapping the reads to their respective genomic positions.

Transcript assembly: Following alignment, the transcript assembly software StringTie was used to construct transcript models with the aligned reads and detect and quantify RNA molecules.

Annotation-based classification: The transcripts were compiled and annotated using RefSeq databases, which include data pertaining to annotated mRNA, lncRNA, and circRNA sequences. Transcripts that overlapped with the annotated mRNA, lncRNA or circRNA regions were categorized as mRNAs, lncRNAs or circRNAs, respectively.

Feature-based classification: The transcripts were also classified according to their structural features.

Quantitative analysis: After classification, the abundance of each RNA type (mRNA, lncRNA, or circRNA) was quantified by counting the number of reads mapped to each transcript using HTSeq.

Normalization and differential expression analysis: The FPKM normalization method was used to compare expression levels between different RNA types or between samples. Differential expression analysis was then performed.

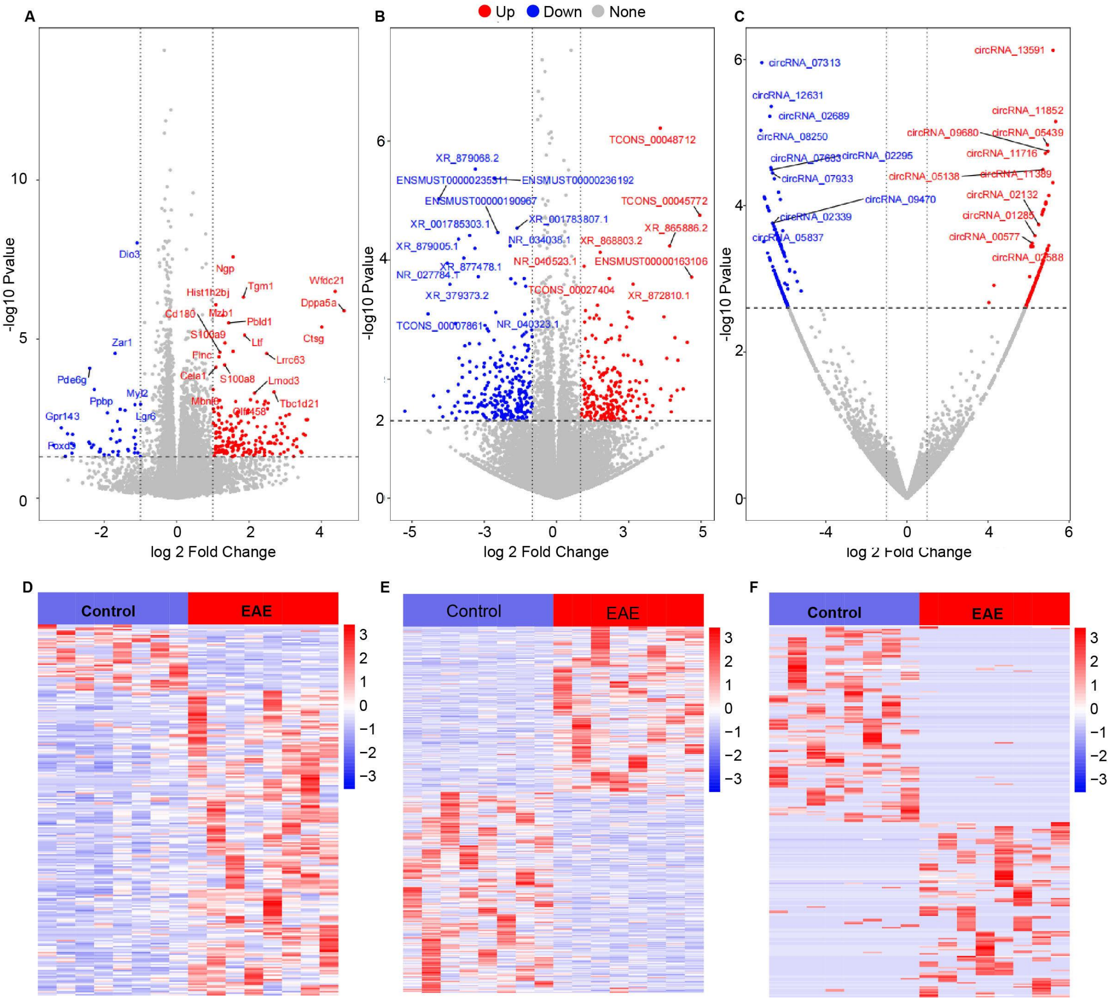

Figure S3. Differential expression analysis of the self-generated RNA-seq dataset (EAE mice brain-GSE253318).

Volcano plots and heatmaps of the differentially expressed RNAs between the EAE samples and controls. (A, D) mRNAs, (B, E) lncRNAs, and (C, F) circRNAs. Red represents high expression, and blue represents low expression. The heatmaps show the relative expression values.

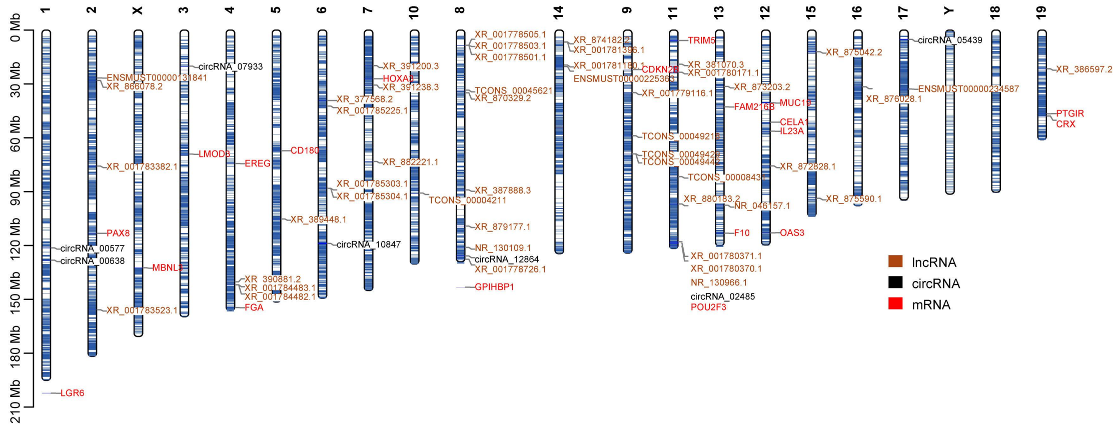

Figure S4. Chromosomal locations of differentially expressed mRNAs, lncRNAs and circRNAs in the ceRNA network of self-generated RNA-seq dataset (EAE mice brain-GSE253318).

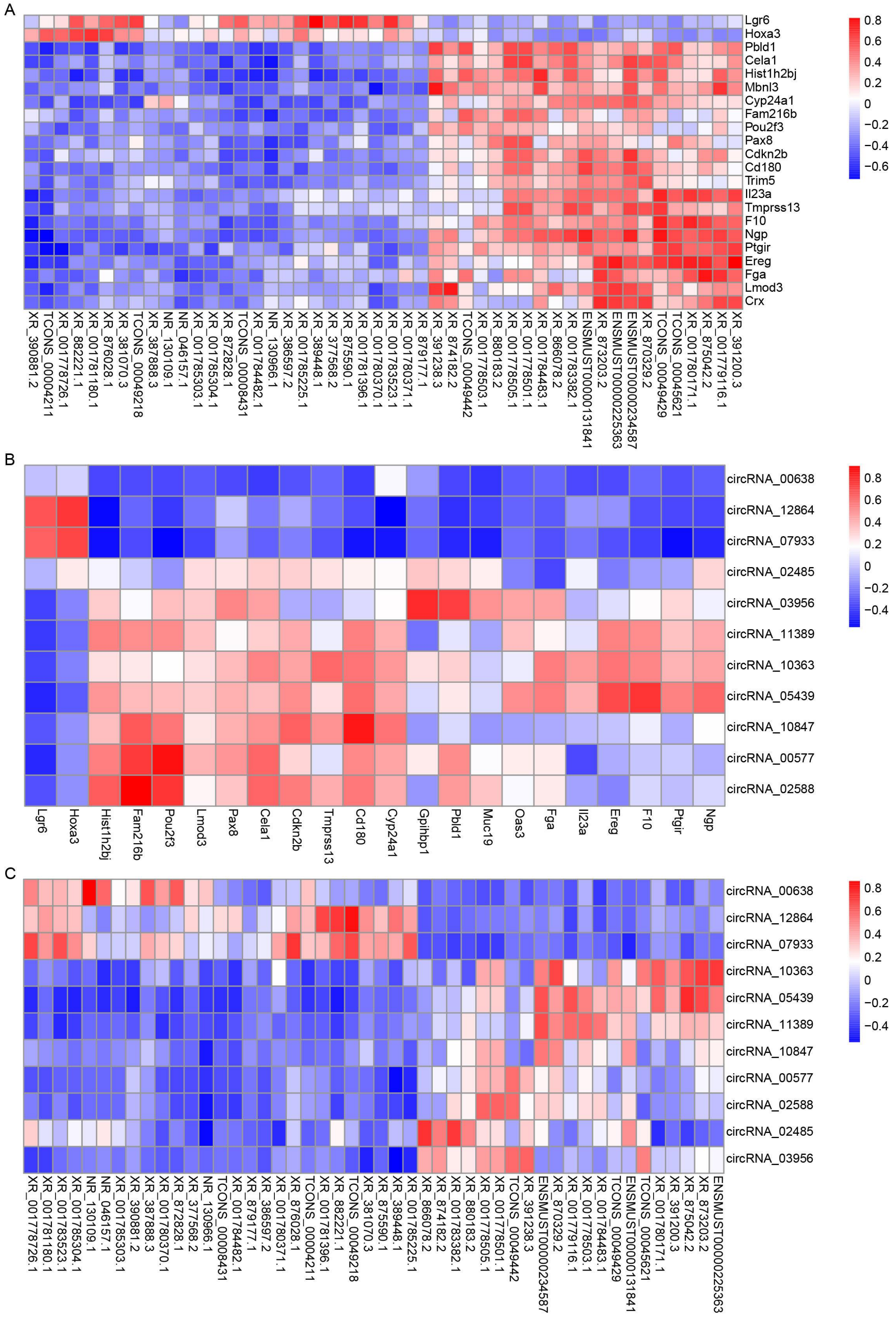

Figure S5. Correlation analysis of the hub genes in the ceRNA network of self-generated RNA-seq dataset (EAE mice brain-GSE253318).

(A) Heatmap of the correlations between DEmRNAs and DElncRNAs. (B) Heatmap of the correlations between DEmRNAs and DEcircRNAs. (C) Heatmap of the correlations between DElncRNAs and DEcircRNAs. Blue indicates negative correlations; red indicates positive correlations. Heatmap values are normalized to z-scores.

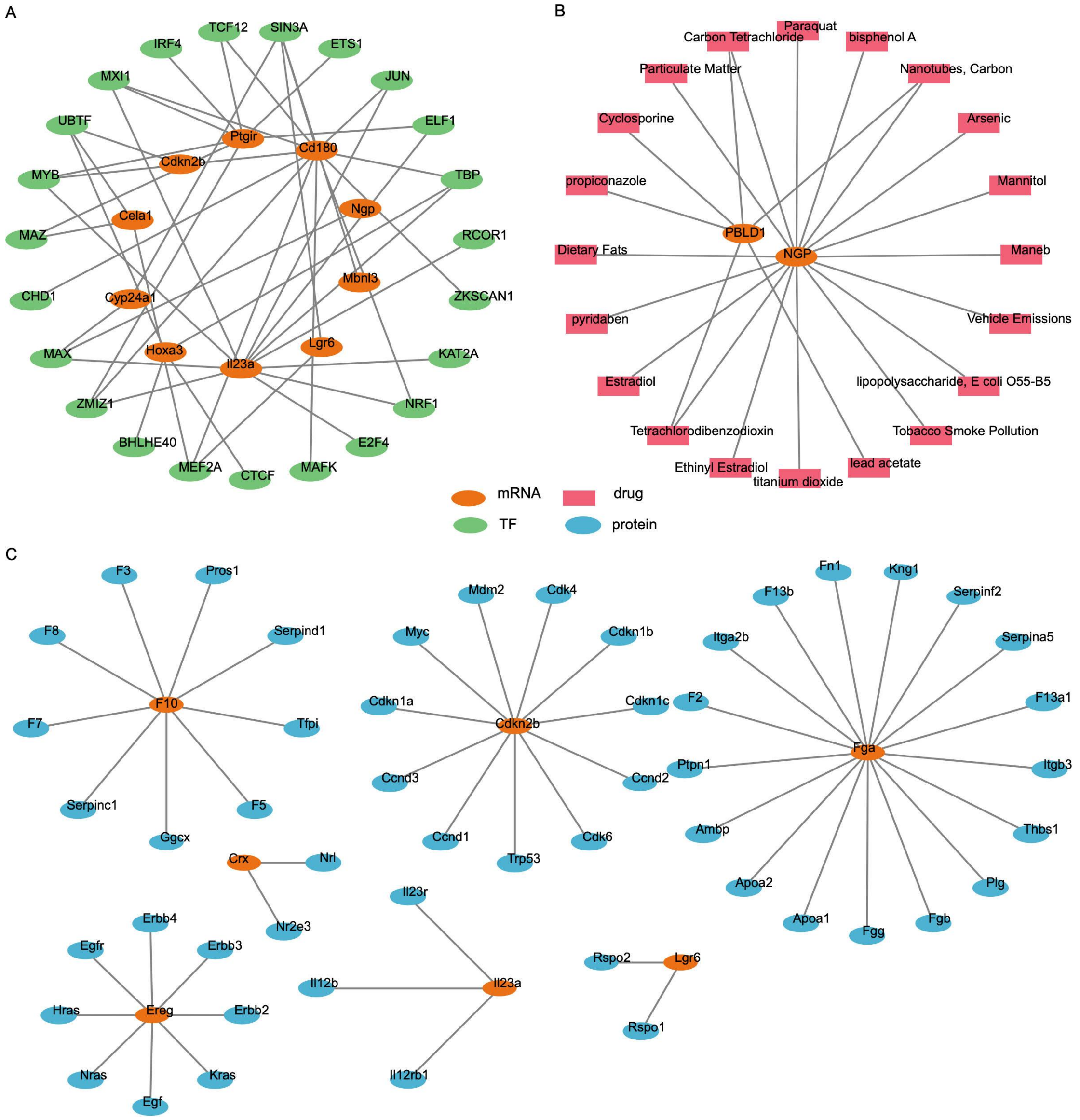

Figure S6. Interaction network analysis of DEmRNAs in the ceRNA network of self-generated RNA-seq dataset (EAE mice brain-GSE253318).

(A) DEmRNA-transcription factor (TF) interaction network. (B) DEmRNA-small-molecule drug interaction network. (C) DEmRNA-RNA-binding protein (RBP) interaction network.

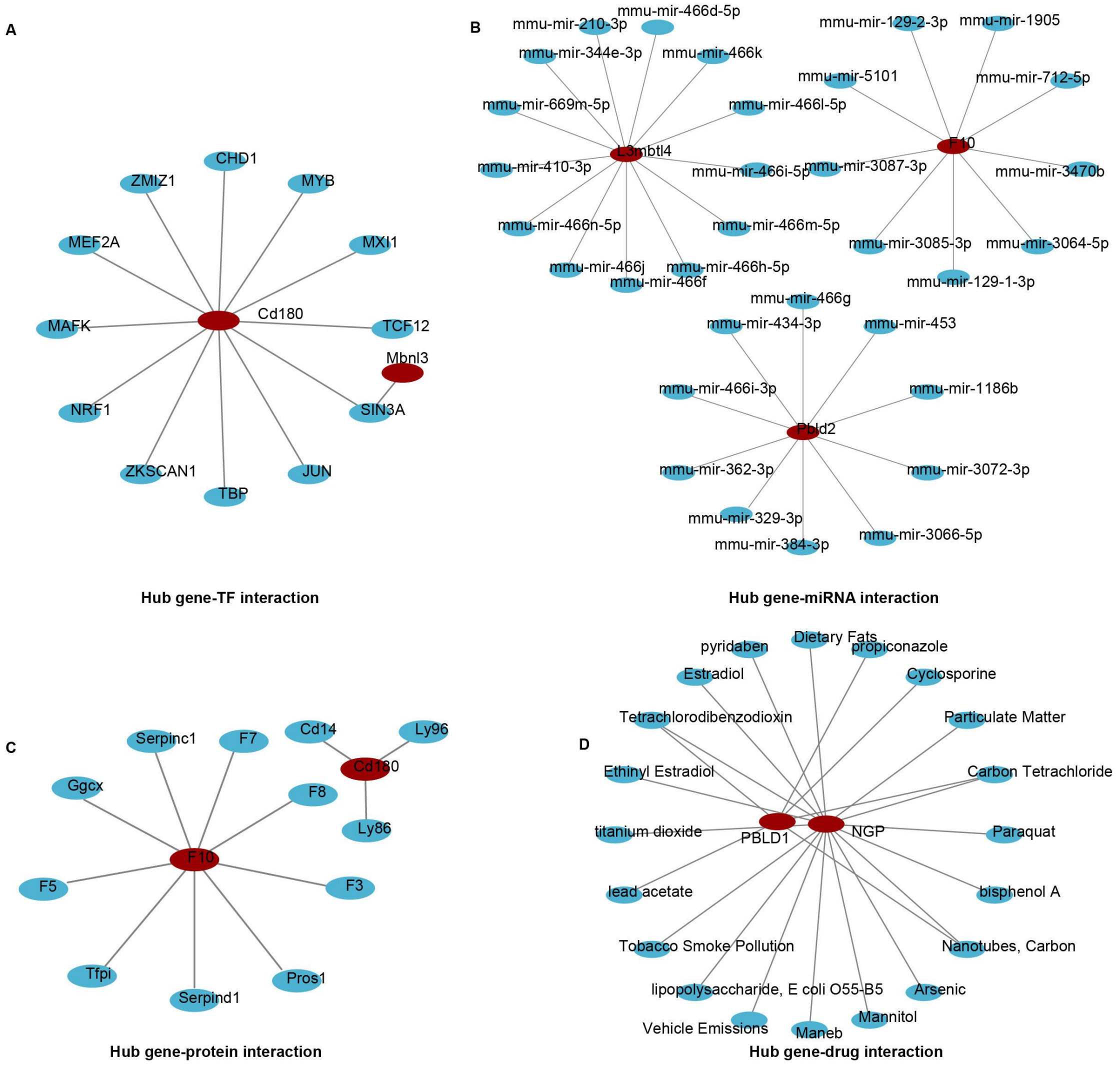

Figure S7. Network analysis of the six signature genes in self-generated RNA-seq dataset (EAE mice brain-GSE253318).

Predicted interaction network analysis of the (A) DEmRNAs and TFs, (B) DEmRNAs and miRNAs, (C) DEmRNAs and RBPs, and (D) DEmRNAs and small-molecule drugs.

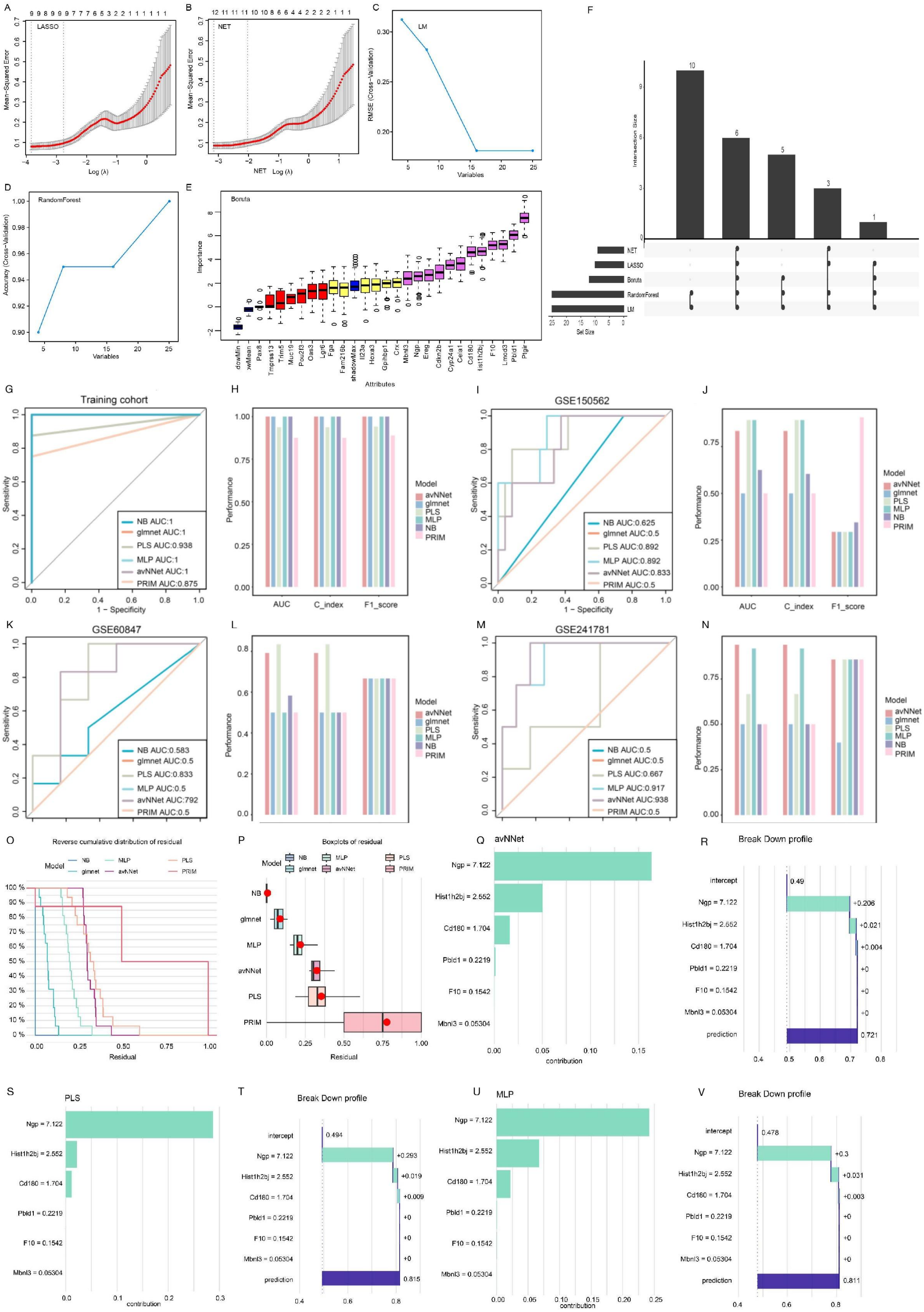

Figure S8. Integrated machine learning workflow for EAE microglial signature discovery: Feature selection, model validation, and interpretability.

(A-F) Feature selection using five ML algorithms for self-generated RNA-seq dataset (EAE mice brain-GSE253318): (A) LASSO coefficient trajectories for feature selection; (B) Elastic net regularization path highlighting key biomarkers; (C) Linear

regression coefficients of prioritized genes; (D) RF importance scores of differentially expressed mRNAs (DEmRNAs); (E) Boruta algorithm output showing 12 significant DEmRNAs (purple bars, $p < 0.05$) and their importance scores (Y-axis); (F) Consensus biomarkers identified across all five algorithms.

(G-N) Model validation across training and external cohorts: (G, H) ROC curves, AUCs, C-indexes, and F1 scores for six ML models (NB, MLP, avNNet, PLS, PRIM) in the self-generated RNA-seq dataset (EAE mice brain-GSE253318). (I, J) Validation on the EAE mice spinal cord-GSE150562 dataset; (K, L) Validation on the EAE mice spinal cord-GSE60847 dataset; (M, N) Validation on the EAE & cuprizone mice brain-GSE241781 dataset. Overlapping ROC curves indicate comparable performance between models.

(O-V) Model interpretability and error analysis: (O) Inverse cumulative residual distribution curves assessing prediction errors across models; (P) Box plots of residual distributions (median ± IQR); (Q, R) SHAP values (Q) and directional contributions (R) of six DEmRNAs in the avNNet model; (S, T) SHAP values (S) and contributions (T) in the PLS model; (U, V) SHAP values (U) and contributions (V) in the MLP model.

ROC, receiver operating characteristic; AUC, area under the curve; SHAP, SHapley Additive exPlanations; LASSO, least absolute shrinkage and selection operator; RF, random forest; NB, naive Bayes; MLP, multilayer perceptron; avNNet, model-averaged neural network; PLS, partial least squares; PRIM, patient rule induction method; SHAP: SHapley Additive exPlanations.

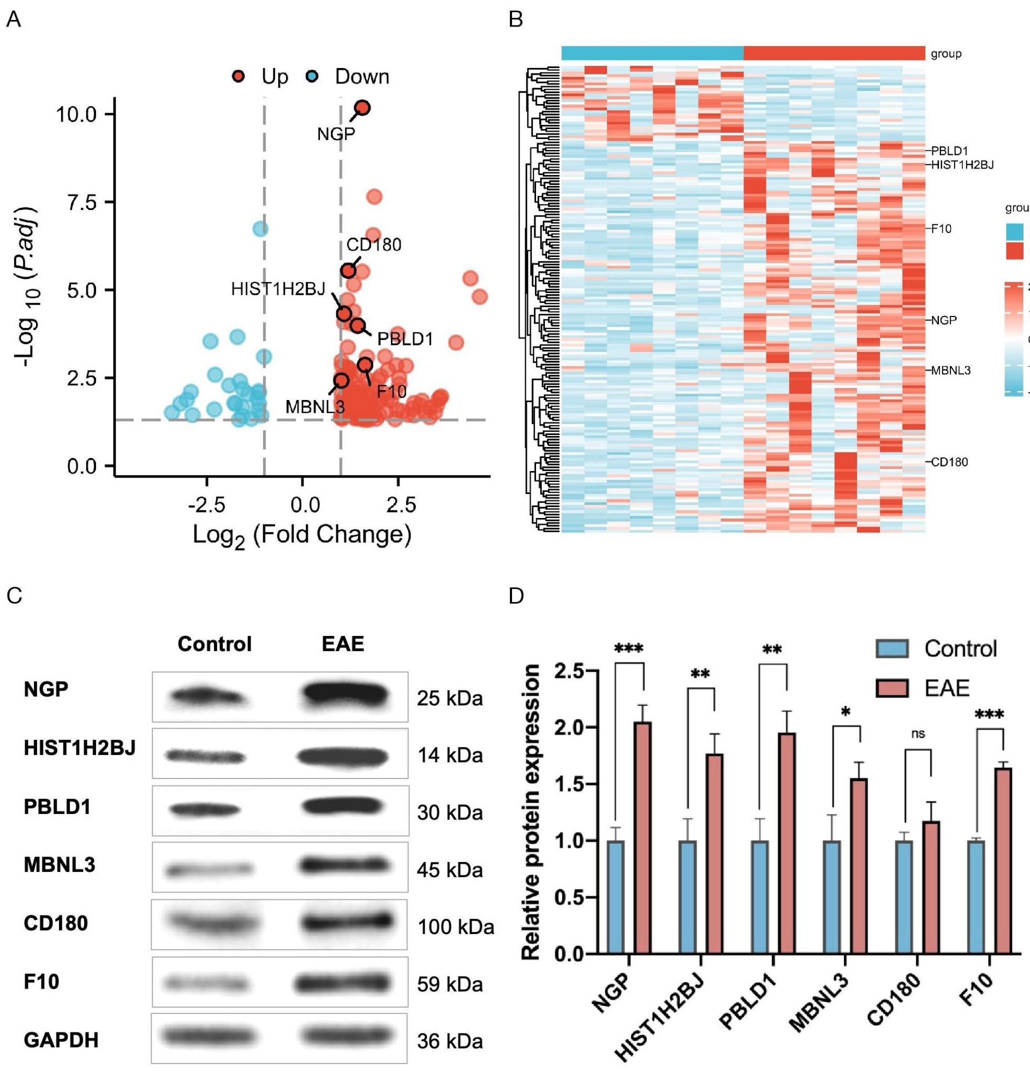

Figure S9. Validation of key gene and protein expression in EAE microglia.

(A) Volcano plot of differentially expressed genes (DEGs) in EAE microglia versus controls. Red dots denote significantly upregulated genes (p < 0.05); blue dots indicate downregulated genes. (B) Hierarchical clustering heatmap of six candidate genes. Color scale reflects z-score-normalized expression (red: high; blue: low). (C) Western blot analysis of protein levels for NGP, HIST1H2BJ, PBLD1, MBNL3, CD180, and F10, with GAPDH as a loading control. Molecular weight markers (kDa) are shown. (D) Densitometric quantification of Western blot bands normalized to GAPDH (mean ± SEM, *p < 0.05, **p < 0.01, **p < 0.001 vs. control; two-way ANOVA with Tukey's post hoc test).

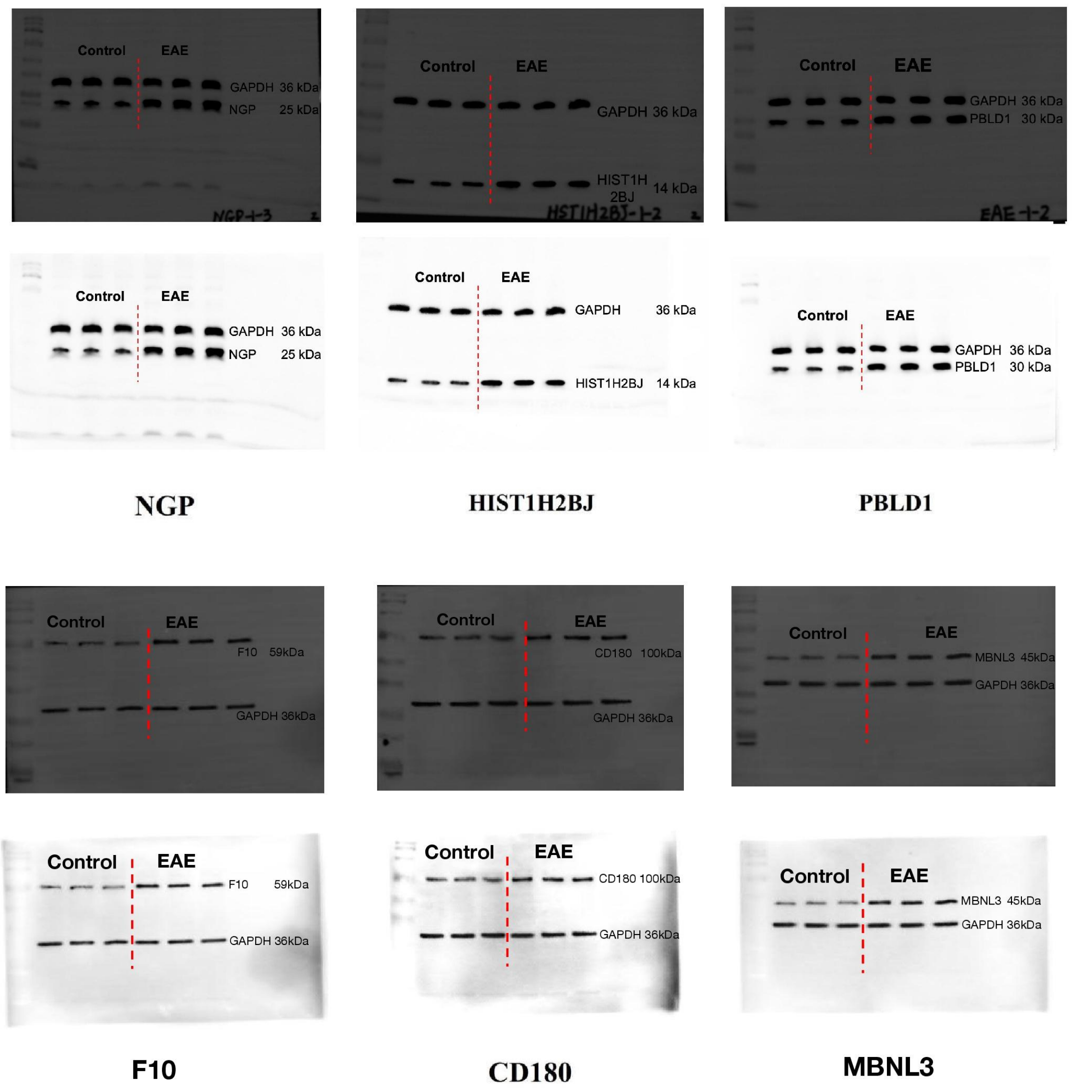

Figure S10. Original Western blot membranes of NGP, HIST1H2BJ, MBNL3, CD180, and F10 in EAE (Experimental Autoimmune Encephalomyelitis) and control microglia, with GAPDH as the loading control. Molecular weight markers (kDa) are indicated on the right.
